# Neuropathological spectrum of anti-IgLON5 disease and stages of brainstem tau pathology: updated neuropathological research criteria of the disease-related tauopathy

**DOI:** 10.1007/s00401-024-02805-y

**Published:** 2024-10-14

**Authors:** Ellen Gelpi, Raphael Reinecke, Carles Gaig, Alex Iranzo, Lidia Sabater, Laura Molina-Porcel, Iban Aldecoa, Verena Endmayr, Birgit Högl, Erich Schmutzhard, Werner Poewe, Bettina Pfausler, Mara Popovic, Janja Pretnar-Oblak, Frank Leypoldt, Jakob Matschke, Markus Glatzel, Elena Maria Erro, Ivonne Jerico, Maria Cristina Caballero, Maria Victoria Zelaya, Sara Mariotto, Anna Heidbreder, Ognian Kalev, Serge Weis, Stefan Macher, Evelyn Berger-Sieczkowski, Julia Ferrari, Christoph Reisinger, Nikolaus Klupp, Pentti Tienari, Osma Rautila, Marja Niemelä, Deniz Yilmazer-Hanke, Mar Guasp, Bas Bloem, Judith Van Gaalen, Benno Kusters, Maarten Titulaer, Nina L. Fransen, Joan Santamaria, Thimoty Dawson, Janice L. Holton, Helen Ling, Tamas Revesz, Liisa Myllykangas, Herbert Budka, Gabor G. Kovacs, Jan Lewerenz, Josep Dalmau, Francesc Graus, Inga Koneczny, Romana Höftberger

**Affiliations:** 1 Division of Neuropathology and Neurochemistry, Department of Neurology, Medical University of Vienna, Waehringer Guertel 18-20, 1090 Vienna, Austria; 2 Comprehensive Center for Clinical Neurosciences & Mental Health Vienna, Medical University of Vienna, Waehringer Guertel 18-20, 1090 Vienna, Austria; 3 Sleep Unit, Neurology Department, Hospital Clinic de Barcelona, IDIBAPS/FCRB, Barcelona, Spain; 4 Institut d’Investigacions Biomèdiques August Pi i Sunyer (FCRB-IDIBAPS), Neuroimmunology Program, Barcelona, Spain; 5 Institut d’Investigacions Biomediques August Pi i Sunyer (IDIBAPS/FCRB), Neurological Tissue Bank of the Biobanc, Hospital Clinic, Barcelona, Spain; 6 Memory Unit, Neurology Department, Hospital Clinic de Barcelona, IDIBAPS/FCRB, Barcelona, Spain; 7 Pathology Department, Biomedical Diagnostic Center, Hospital Clinic de Barcelona-University of Barcelona, IDIBAPS/FCRB, Barcelona, Spain; 8 Department of Neurology, Medical University of Innsbruck, Innsbruck, Austria; 9 Neuro-Critical Care Unit, Department of Neurology, Medical University of Innsbruck, Innsbruck, Austria; 10 Institute of Pathology, Faculty of Medicine, University of Ljubljana, Ljubljana, Slovenia; 11 Department for Vascular Neurology and Intensive Neurological Therapy, University Medical Centre Ljubljana, Ljubljana, Slovenia; 12 Neuroimmunology, Institute of Clinical Chemistry and Laboratory Medicine, Department of Neurology, Kiel University, Kiel, Germany; 13 Institute of Neuropathology, University Medical Center Hamburg-Eppendorf, Hamburg, Germany; 14 Neurology Department, University Hospital Pamplona, Navarra, Spain; 15 Navarra Biomed Research Institute, Pamplona, Spain; 16 Pathology Department, University Hospital Pamplona, Navarra, Spain; 17 Neurology Unit, Department of Neurosciences, Biomedicine, and Movement Sciences, University of Verona, Verona, Italy; 18 Department of Neurology, Kepler University Hospital Linz, and Clinical Research Institute for Neurosciences, Johannes Kepler University, Linz, Austria; 19 Division of Neuropathology, Department of Pathology and Molecular Pathology, Kepler University Hospital Linz, Austria and Clinical Research Institute for Neurosciences, Johannes Kepler University, Linz, Austria; 20 Department of Neurology, Medical University of Vienna, Vienna, Austria; 21 Department of Neurology, St. John’s of God Hospital, Vienna, Austria; 22 Center of Forensic Medicine, Medical University of Vienna, Vienna, Austria; 23 Translational Immunology, Research Programs Unit, Department of Neurology, University of Helsinki, Helsinki University Hospital, Helsinki, Finland; 24 Department of Neurology, Helsinki University Hospital, Helsinki, Finland; 25 Clinical Neuroanatomy, Department of Neurology, University Hospital, Ulm University, Ulm, Germany; 26 Department of Neurology, Donders Institute for Brain, Cognition, and Behaviour, Radboud University Medical Center, Nijmegen, The Netherlands; 27 Department of Neurology, Rijnstate Hospital, Arnhem, The Netherlands; 28 Department of Pathology, Radboud University Medical Centre, Nijmegen, The Netherlands; 29 Department of Neurology, Erasmus University Medical Center, Rotterdam, The Netherlands; 30 Department of Pathology, Amsterdam UMC, Amsterdam, The Netherlands; 31 Department of Pathology, UMC Utrecht, Utrecht, The Netherlands; 32 Queen Square Brain Bank for Neurological Disorders, Department of Neurodegenerative Disease, UCL Institute of Neurology, University College London, London, UK; 33 Neuropathology, Lancashire Teaching Hospitals NHS Foundation Trust, Preston, UK; 34 Department of Pathology, University of Helsinki, HUS Diagnostic Center, Helsinki University Hospital, Helsinki, Finland; 35 Department of Laboratory Medicine and Pathobiology and Tanz Centre for Research in Neurodegenerative Disease, University of Toronto, Toronto, ON Canada; 36 Laboratory Medicine Program and Krembil Brain Institute, University Health Network, Toronto, ON Canada; 37 Department of Neurology, Ulm University Hospital, Ulm, Germany; 38 Department of Neurology, University of Pennsylvania, Philadelphia, PA USA; 39 Institució Catalana de Recerca i Estudis Avançats (ICREA), Barcelona, Spain

**Keywords:** IgLON5, Anti-IgLON5 disease, Anti-IgLON5 tauopathy, Neuropathology, Stages, Brainstem tauopathy, Atypical, TDP-43, Motor neuron disease, ALS, PSP, Dementia, 3R, 4R tau

## Abstract

Anti-IgLON5 disease is a unique condition that bridges autoimmunity and neurodegeneration. Since its initial description 10 years ago, an increasing number of autopsies has led to the observation of a broader spectrum of neuropathologies underlying a particular constellation of clinical symptoms. In this study, we describe the neuropathological findings in 22 patients with anti-IgLON5 disease from 9 different European centers. In 15 patients (68%), we observed a hypothalamic and brainstem-predominant tauopathy of varying severity in which the original research neuropathological criteria were readily applicable. This pathology was observed in younger patients (median age at onset 61 years) with a long disease duration (median 9 years). In contrast, in 7 (32%) patients, the originally described brainstem tauopathy was nearly absent or only minimal in the form of delicate threads, despite mild-to-moderate neurodegenerative features, consistent clinical symptoms and the presence of anti-IgLON5 antibodies in CSF and serum. These patients were older at onset (median 79 years) and had shorter disease duration (median < 1 year). Overall, about one-third of the patients showed concomitant TDP-43 pathology within the regions affected by tau pathology and/or neurodegeneration. Based on these observations and in view of the spectrum of the tau burden in the core regions involved in the disease, we propose a simple staging system: *stage 1* mild neurodegeneration without overt or only minimal tau pathology,* stage 2* moderate neurodegeneration and mild/ moderate tauopathy and *stage 3* prominent neurodegeneration and tau pathology. This staging intends to reflect a potential (age- and time-dependent) progression of tau pathology, supporting the current notion that tau accumulation is a secondary phenomenon related to the presence of anti-IgLON5 antibodies in the CNS. Finally, we adapt the original research criteria of the anti-IgLON5 disease-related tauopathy to include the spectrum of pathologies observed in this larger postmortem series.

**Supplementary Information:**

The online version contains supplementary material available at 10.1007/s00401-024-02805-y.

## Introduction

Anti-IgLON5 disease is currently considered an immune-mediated disorder associated with neurodegenerative changes [49]. The hallmark of the disease is the presence of autoantibodies directed against the cell adhesion molecule IgLON5 in serum and (in > 90%) also in the CSF of affected patients, which is required for establishing the diagnosis of the disease. Experimental studies have shown that anti-IgLON5 antibodies bind and internalize the molecule and alter the neuronal cytoskeletal dynamics leading to secondary neurodegenerative features [33, 34, 48], potentially via acute neuronal hyperactivity [5], and support neuropathological findings of at least a co-segregation with a neurodegenerative tauopathy [23].

As the main targets of the CNS pathology are the hypothalamus and particularly the brainstem, patients frequently present with prominent signs of bulbar dysfunction along with a characteristic sleep disorder with both NREM and REM parasomnias, stridor, and sleep apnea [19, 49]. All levels of the brainstem may be affected and there appears to be a caudo-cranially decreasing gradient of severity of pathology, from the tegmentum of the medulla oblongata upwards to the tegmentum of the pons, midbrain, and downwards to the upper cervical cord including anterior and posterior horns. There is also prominent involvement of the hypothalamus; the hippocampus can be variable affected, but there is only minimal involvement of the basal ganglia and no obvious involvement of cortical regions [23]. The tegmentum of the medulla oblongata and the pons are one of the main regulators of REM sleep through anatomical and functional circuitries from and between the dorsomedial medulla, the subcoeruleus region, reticular formation, raphe, amygdala and hippocampus [51]. Bulbar symptoms such as sleep apnea, stridor and dysphagia are also explained by the involvement of the different respiratory groups, the nucleus solitarius and the nucleus ambiguus [18, 19].

Since the discovery of the anti-IgLON5 antibodies and a wider availability of serologic tests, the number of identified patients has steadily increased. With this, the clinical spectrum has broadened [7, 26, 29, 35, 40, 55], and in addition to the initial identification of a bulbar syndrome and parasomnia, it has expanded to other symptoms reflecting the involvement of the midbrain and the pons, the hypothalamus, the basal ganglia, and the spinal cord [18, 21, 54]. The type and substrate of cognitive impairment observed in some patients [7, 18] is still unclear. It might be associated with a primary age-related tauopathy (PART)-like pathology observed in the hippocampus in some patients, a cortical dysfunction or dysfunction of basal ganglia circuitries, an alteration of the REM sleep-dependent cognitive functions [51], which are regulated by the dorsomedial medulla oblongata, or a combination of all above. Gaig et al. summarized the most frequent clinical “phenotype mimics” observed so far in anti-IgLON5 disease into progressive supranuclear palsy (PSP) -like, multiple system atrophy-like, acute/subacute encephalitic-like, Huntington’s disease-like, and motor neuron disease-like [17, 18, 21]. Sensory hyperexcitability, possibly due to dorsal horn involvement, and cerebellar ataxia have also been described [13]. Despite this wide range of possible symptoms, the core clinical picture is indicative of severe brainstem dysfunction in most patients. While treatment with immunotherapy (e.g., corticosteroids, immunoglobulins, plasma exchange, and rituximab) has been reported to be either ineffective, or associated with mild improvement of symptoms and prolonged disease stabilization in some patients [27, 30], recent data suggest that there might be indeed a response to immunotherapy if treatment is started early [27, 30].

The original neuropathological description in 2014 of a new hypothalamic and brainstem-predominant neuronal 3- and 4-repeat (R) tauopathy underlying anti-IgLON5 disease included two patients [49], and was followed by 4 additional cases (1 definite and 3 probable), which together set the basis for the establishment of the neuropathological research criteria of the anti-IgLON5 tauopathy in 2016 [23]. These neuropathological criteria aimed to define the characteristic features of the tauopathy, not the disease itself, which is defined by the presence of the autoantibodies and the clinical symptoms. Moreover, they intended to establish a framework for future clinico-pathological and research studies, to define a practical tool for the re-evaluation of archival cases, and particularly for recognizing more patients to define the entire spectrum of the clinico-pathological presentation [23]. As a result, more patients have been identified who underwent autopsy, including archival cases, which are included in the present work. The criteria were also based on a relatively natural course of the disease, as only two of the six first reported patients with postmortem study received corticosteroids. Although most subsequent autopsies fulfilled the originally proposed neuropathological criteria of this characteristic tauopathy, not all did [12, 14]. Particularly, the absence or only minimal presence of phosphorylated tau pathology in the brainstem of some patients with anti-IgLON5 antibodies and symptoms of brainstem dysfunction has challenged the neuropathological classification.

We had the opportunity to re-evaluate previously reported cases and assessed new patients with anti-IgLON5 disease who underwent a neuropathological examination, with the aim to characterize the spectrum of underlying pathologies/proteinopathies. Moreover, as we identified a variation of the tau burden along the core brain regions involved in the disease, we propose a simple staging system potentially reflecting a progression of the pathology along the brainstem and midline structures. We correlate our findings with age, clinical symptoms, disease duration, treatments provided, and genetic risk factors. Finally, we suggest to adapt the diagnostic categories of the original neuropathological research criteria of the anti-IgLON5 disease-related tauopathy according to the new observations in this larger autopsy series. These data may contribute to a better understanding of the disease and provide new information for the interpretation of clinical, prognostic, and/or biomarker data.

## Materials and methods

A total of 22 brains were available for this neuropathological study. Brains were collected by different centers throughout Europe including Austria (*n* = 6), Spain (*n* = 5), Germany (*n* = 3), UK (*n* = 2), Finland (*n* = 2), the Netherlands (*n* = 1), Slovenia (*n* = 1), Italy (*n* = 1), and Denmark (*n* = 1). Ethical approval for the use of postmortem brain tissue for research studies was obtained from the Medical University of Vienna (EK 1454/2018, EK 1636/2019, and EK 1123/2015).

Cases were selected if (1) patients showed consistent clinical features within the spectrum of symptoms reported so far in anti-IgLON5 disease [17, 22] and positive detection of serum/CSF anti-IgLON5 antibodies (*n* = 18; “definite” anti-IgLON5 disease), independently of the neuropathological findings, or (2) the neuropathological examination showed a consistent brainstem tauopathy and adequate clinical information was available, but the anti-IgLON5 antibody status was unknown (4 cases, “probable” anti-IgLON5 disease-related tauopathy) (Table 1; see also suppl Table 1 for the definition of “definite”, “probable” as reported in Ref. [23]). In these cases, the determination of antibodies on formalin-fixed and paraffin-embedded tissue was not possible, and deep-frozen material was not available. We acknowledge that this latter group (*n* = 4) represents a bias towards tauopathy cases, but the intention was to include as many cases as possible to represent the widest possible spectrum of pathologies. Ten cases had been previously published, nine with neuropathological descriptions (Cases #3, #5, #12, #13, #14, #15, #16, #19, #20) [8, 12, 14, 23, 49] and one with clinical aspects only (Case #7) [26].

**Table 1:** Basic demographic and clinical features

Case #	Stage	Sex	Age at onset	Age at death	Disease duration (months)	Cause of death	Presentation	Clinical subtype at presentation	Concurrent AI disorder
1	1	Male	81	82	6	Pneumonia, sepsis	Subacute	Bulbar syndrome, sleep syndrome	No
2	1	Male	81	82	11	Respiratory insufficiency (unknown cause)	Subacute	Movement disorder, cognitive impairment, epilepsy	Yes, asthma
3	1	Male	71	73	24	Sudden death (unknown cause)	Chronic	Sleep disorder, PSP-like syndrome	Unknown
4	1	Male	85	86	9	Urinary infection and sepsis	Subacute	Sleep disorder	No
5	1 + mild TDP	Female	69	70	15	Autonomic dysfunction, bradycardia	Subacute	Sleep disorder, cognitive impairment	No
6	1 + TDP	Male	77	77	9	Pneumonia	Subacute	Bulbar symptoms, PSP-like syndrome	No
7	1	Male	76	78	26	Probable sepsis (palliative setting)	Subacute	Bulbar syndrome, sleep syndrome	No
8	2	Male	62	75	156	Pneumonia, status epilepticus	Chronic	PSP-like syndrome	No
9	2	Male	75	76	6	Unknown, found dead at home	Subacute	PSP-like syndrome, cognitive impairment	No
10	2 + TDP	Male	66	72	96	Respiratory failure	Chronic	PSP-like syndrome	No
11	2	Male	Likely 69	73	48	Respiratory failure	Subacute/chronic	Bulbar syndrome, ALS-like syndrome	No
12	3	Male	53	59	72	Sudden while asleep	Chronic	Sleep disorder	No
13	3	Male	48	60	144	Respiratory arrest	Chronic	Movement disorder	Unknown
14	3	Male	49	59	120	Respiratory failure	Chronic	Bulbar syndrome	No
15	3	Female	54	66	156	Aspiration pneumonia	Chronic	Sleep disorder, bulbar syndrome	No
16	3	Female	77	87	120	Sudden while asleep	Chronic	Bulbar syndrome	No
17	3	Female	61	70	108	Pneumonia	Chronic	PSP-like syndrome	Yes, rheumatoid arthritis
18	3	Male	50	65	180	Respiratory failures	Chronic	Bulbar syndrome, sleep disorder	No
19	3 (PSP)	Male	67	76	108	Subdural hematoma	Chronic	PSP-like syndrome, bulbar syndrome	No
20	3 + TDP	Female	76	76	6	Sudden while asleep	Subacute	Bulbar syndrome	No
21	3 + TDP	Female	66	71	60	Infection of unknown origin	Chronic	Bulbar syndrome, possible sleep disorder	No
22	3 + TDP	Female	70	76	72	Respiratory failure	Chronic	Bulbar syndrome, sleep disorder	Unknown

Case #	Concurrent cancer disorder	Co-pathologies	Antibody testing	Immunotherapy	CSF analysis	Brain MRI	Video-PSG	HLA type	MAPT haplotype
1	No	ADNC intermediate severity (A3, B2, C2), ARTAG, olfactory alpha-synuclein Lewy body pathology	In-house (TBA/CBA), positive, titer: serum 1:12,800; CSF 1:256	IVIg w/ short time response, RTX	n/a	Generalized atrophy	Severe central sleep apnea with hypoxemic episodes	DRB1*10:01, DQB1*05:01, DRB1*15:01, DQB1*06:02	n/a
2	Yes, multifocal urothelial carcinoma, first diagnosis 2 years before IgLON, relapse 1 year before diagnosis	PART stage III, no ßA4, no alpha-synuclein	In-house (TBA), Euroimmune (CBA), positive (no titer)	Steroids 3 × 500 mg, improvement (mainly cognitive)	8 cells /µl, increased protein (104.5 mg/dl), lactate 3 mmol/l, OCB neg	Global atrophy, microangiopathy	n/a	n/a	n/a
3	Unknown	ADNC intermediate severity (A3, B2, C2)	In-house (TBA/CBA), positive (no titer)	IVIg, no response	Normal	Normal	Abnormal pseudo-rhythmic movements at 0.7 Hz, cramps, and complex movement automatisms during rapid eye movement and non-rapid eye movement phases	DRB1*10:01–DQB1*05:01	H1/H2
4	No	ADNC mild (A1, B1, C0) + mild CAA, microvascular lesions	In-house (TBA/CBA), positive (no titer)	Steroids iv, plasmapheresis, partial and transient improvement	5 cells/µl, increased protein (49 mg/dl)	Non-specific mild small vessel cerebrovascular disease	NREM parasomnia, no REM sleep recorded, slow efficiency, obstructive hypopneas (AHI 27)	DRB1*10:01–DQB1*05:01	n/a
5	No	PART stage III, no ßA4, no alpha-synuclein	Positive (post-mortem)	IVMP, no response	5 cells/µl, normal protein	Normal	Flexion–extension movements throughout, severe central apnea	DRB1*03:01/DRB1*16:01 DQB1*05:02/DQB1*02:01	H1/H1
6	Unknown	ADNC intermediate severity (A2, B2, C1), mild CAA, no alpha-synuclein	Indirect immunofluorescence, positive, titer CSF 1:100, serum 1:1000	No	Normal	Normal	Not performed	DRB1*08:01/DRB1*10:01, DQB1*04:02/DQB1*05:01, HLA-A*02:01/HLA-A*31:01, HLA-B*15:01/HLA-B*40:01, HLA-C*03:03/HLA-C*03:04, DQA1*01:05/DQA1*04:01, DPB1*02:01/DPB1*04:01, HLA-E*01:01/HLA-E*01:03, HLA-F*01:01/HLA-F*01:01, HLA-G*01:01/HLA-G*01:03	H1/H1
7	Yes, metastasized stomach cancer	AGD stage II-III with prominent septal and limbic involvement, NFT in LC	In-house (TBA), Euroimmune (CBA), positive, titer: serum 1:10,000, CSF 1:100	IVMP, oral steroids, RTX	Normal	Global atrophy with emphasis on mesencephalon	No SOREM	n/a	n/a
8	No	AGD limbic, LATE with limbic TDP-43 and early HS, microvascular lesions	In-house (TBA, CBA), positive, titer: serum 1:400, CSF neg	No	Unknown	Atrophy of mesencephalon	Unknown	DRB1*03:01/DRB1*14:01 DQB1*05:03/DQB1*02:01	H1/H1
9	No	ADNC mild (A2, B1, C1), small vessel disease	In-house (TBA, CBA), positive, titer: serum 1:6400, CSF 1:128	IVMP, oral steroids, MMF	Increased protein (77 mg/dl), rest normal	Vascular leukoencephalopathy, general atrophy	Not done	Unknown	n/a
10	No	ADNC mild (A2, B1, C2) + mild CAA, ARTAG, small vessel disease	Euroimmune Biomosaic (IFA, CBA), positive, titer: serum 1:100, CSF 1:10	5 × 1 g IVMP, oral steroid taper, RTX; improvement of consciousness	Normal	Iron accumulation in globus pallidus, dentate nuclei	n/a	DRB1*10:01; DQB1*05:01	n/a
11	Yes,discovered at autopsy: colon carcinoma, silent prostate carcinoma	ADNC mild (A2, B2, C2)	In-house (TBA), positive, (no titer)	No	Normal	Perinatal encephalomalacia, chronic subcortical small vessel disease thalamus, midbrain	Not performed	n/a	n/a
12	No	No ßA4, no TDP-43, no alpha-synuclein	In-house (TBA/CBA), positive (no titer)	Three cycles of intravenous steroids and cyclophosphamide	Increased protein (55 mg/dl), rest normal	Normal	NREM-REM parasomnia	DRB1*10:01, DQB1*05:01	H1/H1
13	Unknown	No ßA4, no alpha-synuclein, LATE (TDP-43 dentate gyrus); PART II	Not done (archival case)	No	Unknown	Unknown	Unknown	Unknown	n/a
14	No	No ßA4, no TDP-43, no alpha-synuclein; PART II	Not done (archival case)	No	Unknown	Generalized atrophy	Unknown	Unknown	n/a
15	No	No ßA4, no TDP-43, no alpha-synuclein; PART II, microvascular lesions	In-house (TBA, CBA), positive, titer: serum 1:3200, CSF 1:32	No	Unknown	Unknown	During episodes: low amplitude slow alpha activity, vertical more than horizontal rapid eye movements, and increased mental and submental muscle tone	DRB1*10:01; DQB1*05:01	H1/H1
16	No	ADNC mild (A1, B1, C1) + mild CAA	Not done (archival case)	No	Increased protein (57 mg/dl), rest normal	Unknown	Unknown	Unknown	n/a
17	Yes, adrenal cortical carcinoma, adenocarcinoma of the lung	No ßA4, no alpha-synuclein; PART II	Indirect immunofluorescence, positive, titer serum 1:3200; no CSF	IVIg with improvement	Unknown	Abnormal, slight parietal cortical atrophy	Unknown	DRB1*01:01/DRB1*04:04, DQB1*05:01/DQB1*03:02, HLA-A*02:01/HLA-A*03:01, HLA-B*07:02/HLA-B*18:01, HLA-C*03:04/HLA-C*07:01, DQA1*01:01/DQA1*03:01, DPB1*04:01/DPB1*04:01, DRB4*01:03, HLA-E*01:01/HLA-E*01:03, HLA-F*01:01/HLA-F*01:03, HLA-G*01:01/HLA-G*01:01	H1/H2
18	No	No ßA4, no alpha-synuclein	Euroimmun (CBA), positive, titer: serum 1:000, CSF 1:10	IVMP, PLEX, RTX with improvement	Mild pleocytosis (15 cells/µL)	Unspecific white matter hyperintensities in the brainstem	Severe insomnia with overall 24 min of sleep, sleep efficiency of 4%, stridor, vocalizations and abnormal movements both during wakefulness and sleep; neither N3 sleep stages nor REM sleep present	DRB1*10:01; DQB1*05:01	H2/H2
19	No	no ßA4, no TDP-43, no alpha-synuclein	In-house (TBA/CBA), positive (no titer)	No	Mildly increased protein (44.7 mg/dl)	Atrophy of the corpus callosum and the midbrain during the course of disease	Unknown	DRB1*01:02, DRB1*03:01; DQB1*02:01, DQB1*05:01	H1/H1
20	No	ADNC mild (A1, B1, C1) + mild CAA	In-house (TBA/CBA), positive (no titer)	Three cycles of intravenous and oral steroids; two cycles of cyclophosphamide	Normal	Normal	NREM-REM sleep parasomnia, stridor	DRB1*10:01, DQB1*05:01	H1/H1
21	No	Intranuclear neuronal hyaline inclusion disease	Not done (postmortem)	No	n/a	Minor cerebellar atrophy, DAT-SPECT: abnormal uptake putamen	n/a	Unknown	n/a
22	Unknown	ADNC mild (A1, B1, C1), ARTAG, no ßA4, brainstem alpha-synuclein Lewy body pathology, small vessel disease	In-house (TBA/CBA), positive (no titer)	2 × IVIg, RTX, IVMP, PLEX	Unknown	Unknown	Unknown	DRB1*01:01, DRB1*11:01, DQB1*03:01 DQB1*05:01, DQA1*01:01, DQA1*05:05	H1/H1

The column “Co-pathologies” represents the summary of associated pathologies observed in the neuropathological examination

*ADNC* Alzheimer’s disease neuropathologic change, *AGD* argyrophilic grain disease, *ARTAG* aging-related tau astrogliopathy, *CAA* cerebral amyloid angiopathy, *CBA* cell based assay, *CSF* cerebrospinal fluid, *HS* hippocampal sclerosis, *IFA* indirect immunofluorescence assay, *IVIg* intravenous immunoglobulins, *IVMP* intravenous methylprednisolone, *LATE* limbic age-related TDP-43 encephalopathy, *NFT* neurofibrillary tangles, *NREM* non-rapid eye movement, *OCB* oligoclonal bands, *PART* primary age-related tauopathy, *PLEX* plasma exchange, *PSG* polysomnography, *PSP* progressive supranuclear palsy, *REM* rapid eye movement, *RTX* rituximab, *SOREM* sleep onset REM, *TBA* tissue based assay

Clinical information was obtained through a structured questionnaire similar to that used in previously reported cases of anti-IgLON5 disease [17]. It includes information about the main symptoms at disease diagnosis and during follow-up, applied treatments, duration of the disease, and cause of death (Table 1, suppl. Table 2). Response to immunotherapy corresponded to a clinically observed, not a patient reported, effect. A subacute presentation was defined when the patient presented a rapid evolution resulting in substantial neurological dysfunction in ≤ 4 months, otherwise the presentation was defined as chronic. Genetic testing for the HLA haplotype was possible in 14 cases, determination of *MAPT* H1/H2 haplotypes and mutational screening was performed in 11 cases [20].

The routine diagnostic neuropathological study was initially performed locally by each contributing center. After personal discussions with the involved partners (in personal meetings, online and/or via electronic correspondence), representative paraffin-embedded tissue blocks and/or unstained paraffin sections were sent to a single center where the majority of autopsies of patients with anti-IgLON5 disease had been examined so far, for centralized evaluation. The minimal required brain regions were those considered to be characteristically affected in the anti-IgLON5 disease-related tauopathy [23] and included at least the brainstem regions midbrain, pons, and medulla oblongata and, if available, one cortical area, the hypothalamus, the hippocampus, and the cerebellum. In those cases where a broad sampling was performed, the following regions and nuclei were evaluated: cortical (frontal, temporal, parietal, occipital cortices), limbic (cingulate, amygdala, hippocampus including dentate gyrus, CA1-CA4 sector, subiculum, entorhinal and transentorhinal cortex), subcortical (caudate nucleus, putamen, external and internal globus pallidus, preoptic area, hypothalamus (paraventricular, ventromedial, dorsomedial, mammillary body), thalamus, zona incerta, nucleus subthalamicus), brainstem (midbrain: inferior colliculi, periaqueductal grey matter, 3rd cranial nerve, red nucleus, s. nigra; pons: pedunculopontine nuclei, dorsolateral tegmental area, locus coeruleus, raphe, pontine base; medulla oblongata: dorsal motor nucleus of the vagal nerve, hypoglossal nucleus, solitary tract and nucleus, n. ambiguus, gigantocellular nuclei/reticular formation, inferior olives), spinal cord (cervical, thoracic, lumbosacral levels, if available, anterior and posterior horns), and cerebellum (vermis, hemisphere, and dentate nucleus).

Evaluation of disease-related protein aggregates was performed with antibodies against phosphorylated tau (clone AT8, pS202/pT205; Thermo Scientific, Rockford, IL, USA), 3-repeat tau isoforms (RD3 Tau, clone 8E6/C11, Millipore, Temecula, CA; USA), 4-repeat tau isoforms (RD4, clone 1E1/A6, Millipore), phosphorylated transactive response DNA binding protein 43 (pTDP-43; clone 11-9, pS409/410; Cosmo Bio, Tokyo, Japan), amyloid βA4 peptide (ßA4; clone 6F/3D; DAKO), alpha-synuclein (clone 5G4; Roboscreen, Leipzig, Germany), p62 (clone 3/p62 lck ligand; BD Transduction Laboratories, Franklin Lakes, NJ, USA), and Fused in Sarcoma/Translocated in LipoSarcoma (FUS; polyclonal, Sigma Aldrich HPA008784, St. Louis, MO, USA). Glial fibrillary acidic protein (GFAP; polyclonal, DAKO, Glostrup, Denmark) and HLA-DR (clone CR3/43, DAKO) immunohistochemistry were performed for the identification of astroglia and microglia, respectively. Neuronal and axonal structures were assessed with anti-neurofilament antibodies (clone SMI31, phosphorylated neurofilaments; clone SMI32, non-phosphorylated neurofilaments; both Sternberger Monoclonal antibodies (SMI), Biolegend Inc. San Diego, CA, USA). Details on the specific stainings performed in the specific brain areas by the participating centers are depicted in supplementary Table 3. All cases were screened for potential *C9orf72* expansion mutations by p62 immunohistochemistry on the cerebellar granular layer and dentate gyrus of the hippocampus [3, 45], and by genetic testing in two cases from Finland.

**Fig. 1:**
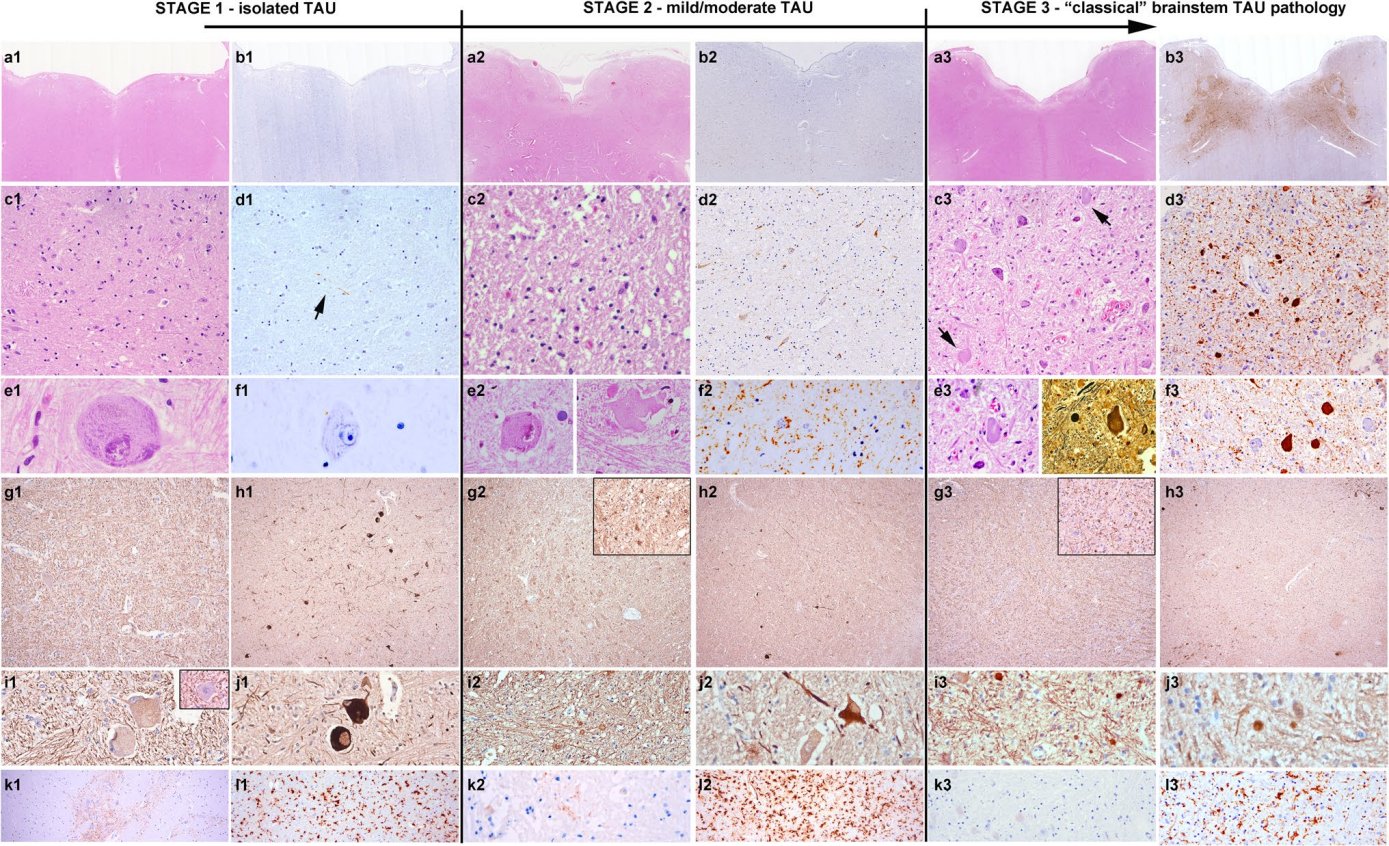
Histological findings at the different stages of pathology. Representative histological images of the neuropathological features of anti-IgLON5 disease at different grades of severity. **a1**–**f1**, **a2**–**f2** and **a3**–**f3**: First row: HE and Tau immunohistochemistry at the level of the medulla oblongata. At low magnification (**a1**–**b3**), extensive tau pathology can be clearly observed in the tegmentum at *stage 3* (**b3**), but not at *stage 1* (**b1**) and barely *at stage 2* (**b2**). Second row: at higher magnification (**c1**–**d3**), Tau pathology is already visible at *stage 2* (**d2**) in form of neuropil threads and pretangles, while at *stage 1* isolated delicate threads are visible (**d1**, arrow). In contrast, HE-stained sections in *stage 1* (**c1**) and *stage 2* (**c2**) already show reactive changes in the reticular formation. In addition, some enlarged neurons are observed at *stage 1* and *stage 2* (**e1**, **e2**), that are tau negative, while at *stage 3*, they are already containing tau-positive neurofibrillary tangles, but are overall reduced in numbers. These enlarged neurons show a slightly increased immunoreactivity for phosphorylated (SMI31; **g1**, **i1**) and particularly of non-phosphorylated (SMI32; **h1**, **j1**) neurofilaments, and have a regular axonal density and morphology. In *stage 2* and *stage 3*, the number of enlarged neurons decreases (**h2**, **j2** and **h3**, **j3**) but an increase in axonal spheroids is detected (**i2**, **i3**). **k1**, **k2**, **k3**: Immmunohistochemistry for IgG4 in the brainstem shows focally marked deposits at stage 1 (k1), mild deposits in single cases at stage 2 (k2) and no deposits at stage 3 (k3). i1, i2, i3: different grades and states of microglial activation at different disease stages (HLA-DR immunohistochemistry)

In the brainstem areas, which were available in all cases, we semiquantified the extent of neuronal loss, astrogliosis, and microglial activation into “mild/moderate” or “severe”. These changes were evaluated on hematoxylin–eosin (HE) and/or immunostained sections, and were assessed by three of the authors blinded to clinical or neuropathological patterns (EG, RH, RR) on a multiheaded microscope. In addition, the burden of tau pathology in specific anatomical regions was assessed semiquantitatively as follows: 0 = absent, *i* = isolated (< 3 tau-positive neuropil threads and /or < 1 pretangle); 1 =  mild (3–15 positive neuropil threads and/or > 2 pretangles and/or < 5 neurofibrillary tangles); 2 = moderate (> 15 positive neuropil threads and/or > 3 pretangles and/or > 5 neurofibrillary tangles); 3 = abundant (abundant neuropil threads and > 10 pretangles and/or tangles) (Fig. 1). The same approach was used for the assessment of pTDP-43 pathology.

The presence and distribution of inflammatory infiltrates was recently described in a subset of patients [8] and has not been specifically evaluated in this study.

Statistical analyses were performed using unpaired two-sided t-test, Fisher’s exact test, ANOVA and Tukey post-hoc test, as well as two-sided Chi-square tests and linear regression with Pearson’s correlation coefficient, where appropriate.

## Results

### Burden of tau pathology

**Fig. 2:**
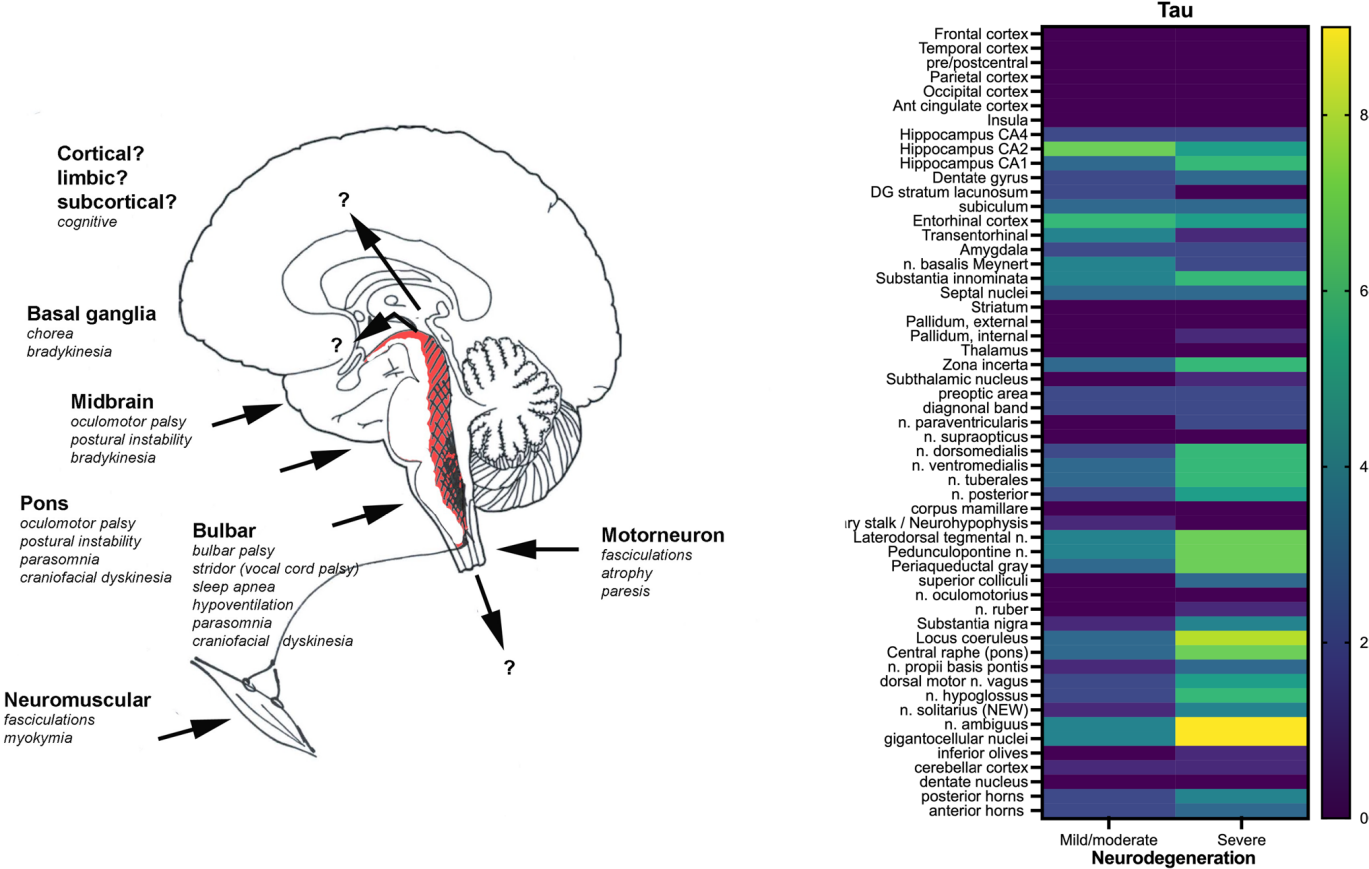
Left: graphical neuroanatomical representation of the main focus of anti-IgLON5 disease neuropathology and possible related clinical symptoms. Right: grouped analysis and heat map; source of variations: tau burden (0 = absent or mild; 1 = moderate or prominent) and neurodegeneration (mild/moderate vs severe) (two-way ANOVA, source of variation: anatomical region: *p* = 0.0004; degree of neurodegeneration: *p* < 0.0001)

**Fig. 3:**
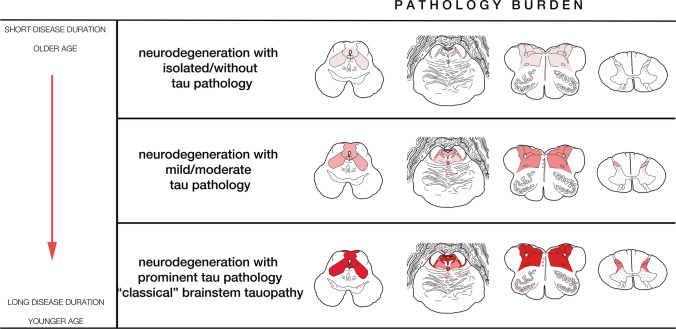
Pathology burden. Graphical representation of the severity of neurodegeneration and tau burden in the brainstem regions observed at the different stages of pathology: midbrain (leftmost), pons (left middle), medulla oblongata (right middle), and upper cervical cord (rightmost), and their correlation with age at onset and duration of disease on the left. Color codes: upper line: the focus of neurodegeneration in the brainstem is represented as a pale-red area with minimal or even absent tau pathology; middle line: the mild-to-moderate deposition of tau is represented in softer red; lower line: prominent tau pathology is represented in intense red and areas with milder or occasional pathology in form of pale-red dots (s. nigra midbrain, pontine base, inferior olives). Figure adapted from the original criteria [23]. Stage and pathology burden. *Stage 1 (upper row)*: mild/moderate neuronal loss/gliosis and isolated tau-positive neuropil threads in the lateral segments of the midbrain and pontine tegmentum and/or reticular formation/respiratory nuclei of the medulla oblongata and/or hypothalamic nuclei/preoptic area/pituitary stalk. *Stage 2 (middle row)*: moderate neuronal loss/gliosis and moderate tau-positive neuropil threads, pretangles and NFT in the previous regions extending to adjacent tegmental nuclei and hypothalamic /prehypothalamic nuclei. *Stage 3 (lower row)*: Prominent neuronal loss/gliosis and extensive tau pathology in the aforementioned regions + mild involvement of s. nigra, inferior olives, dentate nucleus of the cerebellum and glomerula of the granule cell layer of the cerebellar cortex

Among the 22 cases, we observed a variable burden of the tau pathology along the tegmentum of the brainstem, the spinal cord, the hypothalamus/basal forebrain and the hippocampus—regions described in the original neuropathological criteria as being typically affected by the disease. It ranged from nearly absent or minimal/mild in form of isolated threads only visible at high magnification (× 400) (7 cases; 32%), moderate with a higher density of neuropil threads already identified at lower magnification (× 100) and the presence of a low density of pretangles and/or neurofibrillary tangles (4 cases; 18%), to prominent with a high density of neuropil threads, pretangles and/or tangles (15 cases; 50%). Details of the burden and topographical distribution of the tau pathology in the individual anatomical regions are shown in Figs. 1, 2 and Table 2. The neurodegenerative changes involving the brainstem tegmentum were variable and increased with increasing tau burden, but were observed in all cases, including those with absent or mild tau pathology (Figs. 1, 3).

**Table 2:**
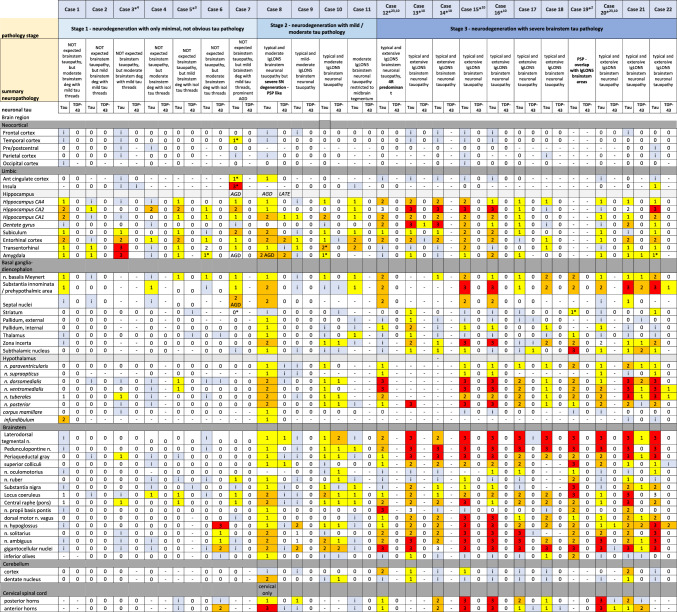
Heatmap of the tau and TDP-43 pathology load in different neuroanatomical regions for each case

Based on the following semiquantitative assessment:

– = not available; 0 (white) = absent, i (pale blue) = isolated (< 3 tau-positive neuropil threads and /or < 1 pretangle); 1 (yellow) = mild (3–15 positive neuropil threads and/or > 2 pretangles and/or < 5 neurofibrillary tangles); 2 (orange) = moderate (> 15 positive neuropil threads and/or > 3 pretangles and/or > 5 neurofibrillary tangles); 3 (red) = abundant (abundant neuropil threads and > 10 pretangles and/or tangles)

Abbreviations: *PAG* periaqueductal grey matter, *FR* reticular formation, *CN* cranial nerve, *LC* locus coeruleus, *SN* substantia nigra, *DMNV* dorsal motor nucleus vagal nerve, *MN* motor neurons, *AH* anterior horn, *SGel* substantia gelatinosa, *GFA* granular fuzzy astrocyte, *CB* coiled body, *ADNC* Alzheimer’s disease neuropathological  change, *PART* primary age-related tauopathy, *NFT* neurofibrillary tangles, *CAA* amyloid angiopathy, *AGD* argyrophilic grain disease, *LATE* limbic age-related TDP-43 encephalopathy, *HS* hippocampal sclerosis, *ARTAG* aging-related tau astrogliopathy

*Includes glial pathology: granular fuzzy astrocytes (GFA) in the grey matter and/or thorn shaped astrocytes in the glia limitans (TSA)

### Identification of different stages of pathology

Detailed topographical mapping and grouped analysis showed a progressive increase in the tau pathology load along the brainstem and hypothalamic regions (Fig. 2, Table 2). Based on these findings, and the experimental data supporting that the tauopathy is a secondary event of the autoimmune disorder [33, 34, 48], we suggest a classification into three pathological stages of the anti-IgLON5 disease-related tauopathy. These stages showed a positive correlation with disease duration and an inverse relationship with age at onset (see below).

**(I)** ***Stage 1*** **:** **brainstem degeneration without overt or minimal tau pathology**

*Stage 1* was observed in 7/22 (32%) patients. This stage is characterized by a mild-to-moderate gliosis and microglial activation in the anterior hypothalamus and brainstem tegmentum, particularly in the periaqueductal grey matter, lateral tegmentum, reticular formation and respiratory nuclei of the medulla oblongata (solitary nucleus and n. ambiguus). Enlarged neurons may be observed in the reticular formation (Fig. 1e1). Tau pathology may be absent or is minimal and presents in form of isolated tau-positive cell processes/neuropil threads in these regions (Fig. 1d1). The infundibulum may show mild-to-moderate tau-positive threads (suppl. Fig. 1). In some patients, the hippocampus may be affected by tau pathology in a type and distribution similar to PART, but with more frequent involvement of the CA2 sector and the dentate gyrus. The cortical areas, striatum, lenticular nucleus, n. subthalamicus, thalamus and cerebellum are devoid of tau pathology.

**(II)** ***Stage 2*** **:** **brainstem degeneration with mild/moderate tau pathology**

This grade of pathology was observed in 4/22 cases (18%). In this stage, gliosis and microglial activation in the anterior hypothalamus and prehypothalamic region, as well in the brainstem tegmentum are more prominent than in the previous stage, as well as the burden of the tau pathology. It involves additional regions of the basal forebrain, particularly the substantia innominata, and of the tegmentum of the brainstem, including the laterodorsal tegmental region, pedunculopontine nuclei, and locus coeruleus of the pons, and the pars reticulata of the substantia nigra in the midbrain. The medulla oblongata shows more prominent tau pathology in the aforementioned regions, in addition to a moderate involvement of the dorsal raphe and of the dorsal motor nucleus of the vagal nerve and the motor nucleus of the hypoglossus. In addition to clusters of neuropil threads, a low amount of pretangles and neurofibrillary tangles may be already observed (Fig. 1d2, f2). These may be also identified in the dorsal and anterior horns of the spinal cord (suppl. Fig. 2). In the cerebellar cortex a fine granular synaptic immunoreactivity pattern may be detected in the synaptic glomerula of the granule cell layer (suppl. Fig. 1). The dentate nucleus is usually not or only minimally involved. The cortical areas and the striatum are practically devoid of tau pathology, while the thalamus, n. subthalamicus and zona incerta may show mild tau-positive threads and isolated pretangles and/or tangles. The pars reticulata more than the pars compacta of the substantia nigra show at the most a moderate tau pathology.

**(III)** ***Stage 3*** **: brainstem degeneration with prominent tau pathology—“classical” pattern**

*Stage 3* of prominent tau burden was observed in 11/22 cases (50%), and included the six original cases on which initial neuropathological criteria were based on and five additional cases. The pattern was very homogeneous among these cases and was readily identifiable.

At this stage of pathology, all areas reported in the original description of the anti-IgLON5 disease-related tauopathy are severely affected and show high burden of tau pathology (Figs. 1b3, d3, f3, 3, Table 2). The key regions include the prehypothalamic region, anterior hypothalamic nuclei, substantia innominata, n. basalis Meynert (moderate involvement), zona incerta, periaqueductal grey matter, laterodorsal tegmental area, pedunculopontine nuclei, locus coeruleus, raphe, dorsal motor nucleus of the vagal nerve, n. hypoglossus, and reticular formation. In some cases, the pontine base may show a variable amount of pretangle and/or tangles. The inferior olives may show single neurofibrillary tangles and perineuronal neuritic clusters. The anterior and posterior horns of the spinal cord show variable amounts of tau pathology (suppl. Fig. 2). In the cerebellum, tau positivity in the synaptic glomerula of the granule cell layer is usually focal, single Cajal cells may show a cytoplasmic immunoreactivity, while Purkinje cells and their apical dendrites remain mostly negative (suppl. Fig. 1). The dentate nucleus shows single tangles/pretangles/threads. The cortical areas remain devoid of pathology, while the striatum and thalamus may show at most single pretangles and/or tangles and/or neuropil threads. At this stage, the tau pathology in the hippocampus is variable but appears more extensive than in *stage 1* and *stage 2* in some cases, and involves more neurons of the CA1 sector and the ento- and transentorhinal regions. Although the involvement of the dentate gyrus and the CA2 sector is frequently observed in the anti-IgLON5 disease-related tauopathy, a clear delineation from PART pathology may be not possible and the presence of associated pathologies, like argyrophilic grain disease, pose a difficulty in the interpretation of hippocampal pathology.

### Distribution of tau isoforms

In eight cases both, 3R and 4R tau isoforms contributed to the tau inclusions in the brainstem, which were mainly neuronal but could also include some oligodendroglial coiled-body type inclusions in the brainstem [23] and some ramified astrocytes in the limbic system. In three cases, however, there was a predominance of 4R tau isoforms. One case was retrospectively identified by reviewing the clinical information of a patient who had died as a result of a PSP-like syndrome with atypical features that were suggestive of anti-IgLON5 disease. Archived CSF confirmed the presence of anti-IgLON5 antibodies and the neuropathological examination showed an extensive brainstem tauopathy, as expected for the anti-IgLON5 disease-related tauopathy, which, however, formally fulfilled the neuropathological criteria of a PSP phenotype [8]. This included the presence of > 1 tufted astrocytes in the perirolandic region or the putamen, in this case in the putamen, and > 1 4R tau-positive neurofibrillary tangles in the globus pallidus, n. subthalamicus and s. nigra (two of the three regions), in addition to frequent coiled bodies, as reported [8]. Nevertheless, there was a clear brainstem predominance of the pathology which involved also the hypothalamic and brainstem nuclei affected in the other cases of anti-IgLON5 disease-related tauopathy at *stage 3* with a mixture of 3R + 4R tau isoforms. This case also showed focal tau positivity in the synaptic glomerula of the cerebellar granule cell layer. Another case with a predominance of 4R tau isoforms was included in the very first description of the disease [49]. Finally, the third case (#8, clinical features consistent with a PSP-like phenotype and positive anti-IgLON5 antibodies), presented typical distribution of the anti-IgLON5 disease-related tau pathology with mainly neuronal tau pathology, which was, however, dominated by 4R tau isoforms, and showed no astrocytic tau pathology definitory of any other known 4R tauopathy. Interestingly, this case had a severe nigral degeneration with disproportionally low (mild-moderate) tau pathology in this region, and showed also some involvement of the pontine base and the dentate nucleus of the cerebellum, as can be also observed in PSP. However, there was no involvement of the basal ganglia or cortical regions. In the pallidum, an area of necrosis/infarct in the context of extensive small vessel disease was also observed.

**Fig. 4:**
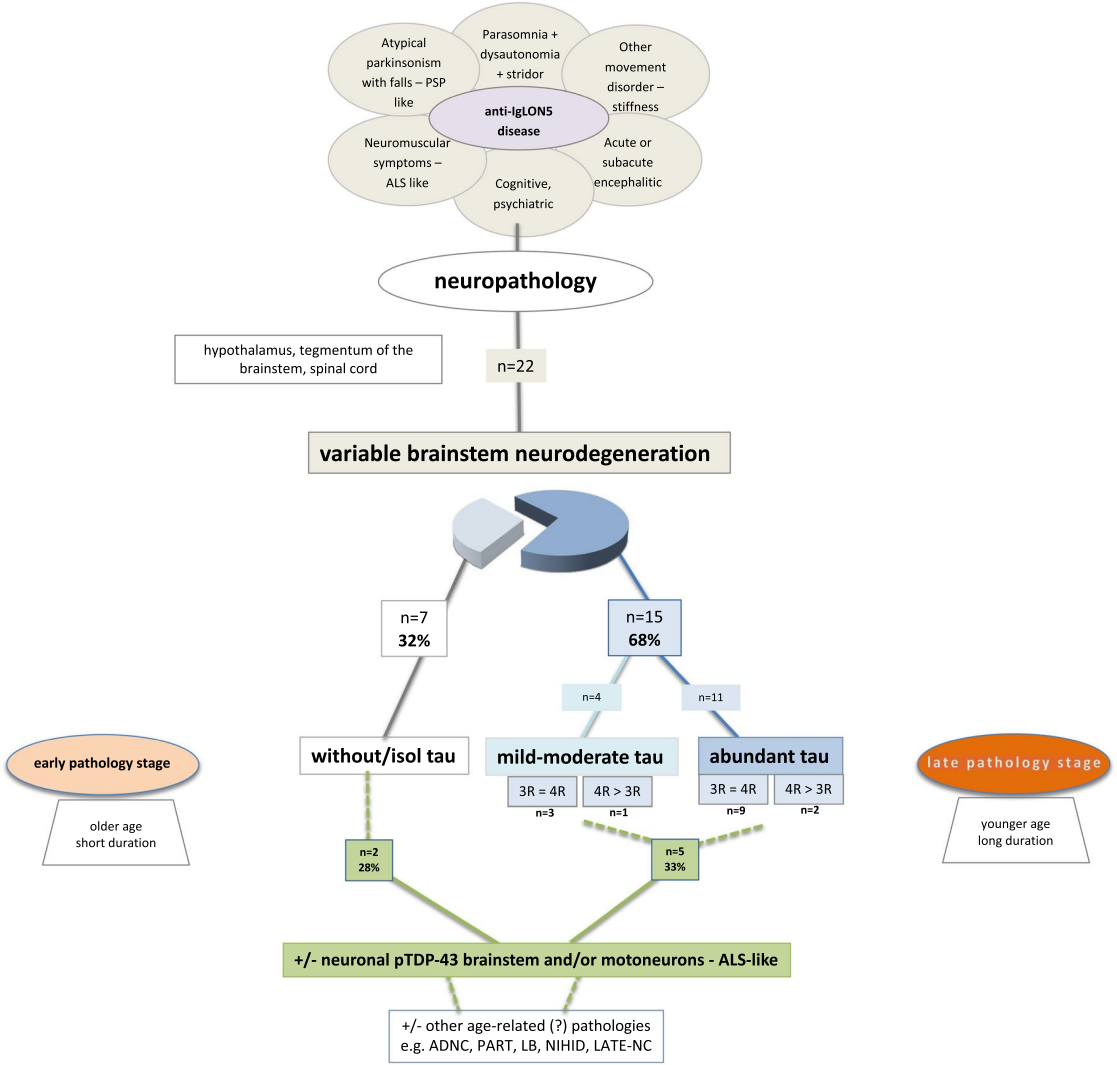
Summary of the neuropathological findings in 22 patients with anti-IgLON5 disease. Of these, 68% showed a brainstem tauopathy, while 32% did not. The latter group was represented by older patients with shorter disease duration, compared to the patients with brainstem tauopathy, who were younger at disease onset and had longer disease duration

A graphical summary of the main findings in the 22 patients is represented in Fig. 4.

### Concomitant pathologies

(I) TDP-43 pathology in the brainstem and spinal cord

TDP-43 pathology in the brainstem and spinal cord was observed in 7/22 cases (32%): 5 had associated brainstem tau pathology (2 at *stage 2* and 3 at *stage 3***)** and 2 had minimal or no tau pathology (at *stage 1*) (suppl. Fig. 2). TDP-43 inclusions were most frequently identified in motor and non-motor neurons of the brainstem and spinal cord and/or in areas affected by tau pathology, i.e., the tegmentum medullaris, including reticular formation and the anterior horns of the spinal cord (suppl material in Ref. [49]). The morphology of the aggregates in motor neurons included skein-like inclusions and a fine granular diffuse cytoplasmic staining (suppl. Fig. 2j, k). Single Bunina bodies were also identified in motor neurons on HE-stained sections in single cases (e.g., case #4, case #20) (suppl. Fig. 2f), but no pale or Lewy-like inclusions were identified. Moreover, axonal spheroids in the anterior horn of the spinal cord were present in single cases with prominent motor neuron involvement (case #18) (suppl. Fig. 2d). In addition, few coiled-body- like TDP-43 inclusions were observed in oligodendrocytes (supp. Fig. 2l). Compact neuronal inclusions were also detected in the reticular formation. Minimal signs of corticospinal degeneration were observed in single cases (case #20), but without obvious TDP-43 inclusions in upper motor neurons. Neurons of the 3rd and 6th cranial nerves were also involved.

(II) Other associated pathologies

Other associated pathologies were identified in 74% of cases, including common findings in apparently neurologically unimpaired aged persons, like low to moderate density of ßA4-amyloid deposits in form of diffuse and/or compact plaques, few alpha-synuclein aggregates, limbic TDP-43 pathology, argyrophilic grain pathology, mild-to-moderate ento/transentorhinal and hippocampal tau pathology, and vascular lesions. Co-pathologies were detected in both, patients with and without the brainstem tauopathy (Table 1). A single case had, in addition to the anti-IgLON5 disease-related brainstem tauopathy and TDP-43 aggregates in brainstem motor neurons as also seen in ALS, frequent intranuclear hyaline inclusions in an extent and distribution that were consistent with neuronal intranuclear hyaline inclusion body disease (NIHID) [25]; genetic testing to exclude fragile-X tremor ataxia syndrome or *NOTCH2NLC* gene alterations for NIHID was not possible. In two elderly patients, we found concomitant ßA4-amyloid pathology, and aging-related tau astrogliopathy (ARTAG), respectively; in two other cases, we identified incidental Lewy body pathology [8]; and in further two patients, we detected argyrophilic grain pathology. No FUS aggregates were detected in any of the cases. Small vessel disease and an old lacunar infarction was identified in the basal ganglia of the patient in which the brainstem tauopathy was dominated by 4R tau isoforms. This patient had in addition neuropathological changes consistent with argyrophilic grain disease (AGD) and limbic age-related TDP-43 encephalopathy (LATE) associated with incipient hippocampal sclerosis (patient #8, Table 1).

In those cases, in which other additional/co-existing pathologies are found independently of a “brainstem/motor neuron predominant TDP-43 pathology”, we suggest reporting them preliminarily as additional pathologies, as long as evidence for a strong association with anti-IgLON5 disease is lacking (and co-incidentality, age-related changes, vascular disease, or other mechanisms cannot be excluded).

### Clinicopathological correlations (Table 3, Figs. 5, 6, suppl. Fig. 3–6)

**Fig. 5:**
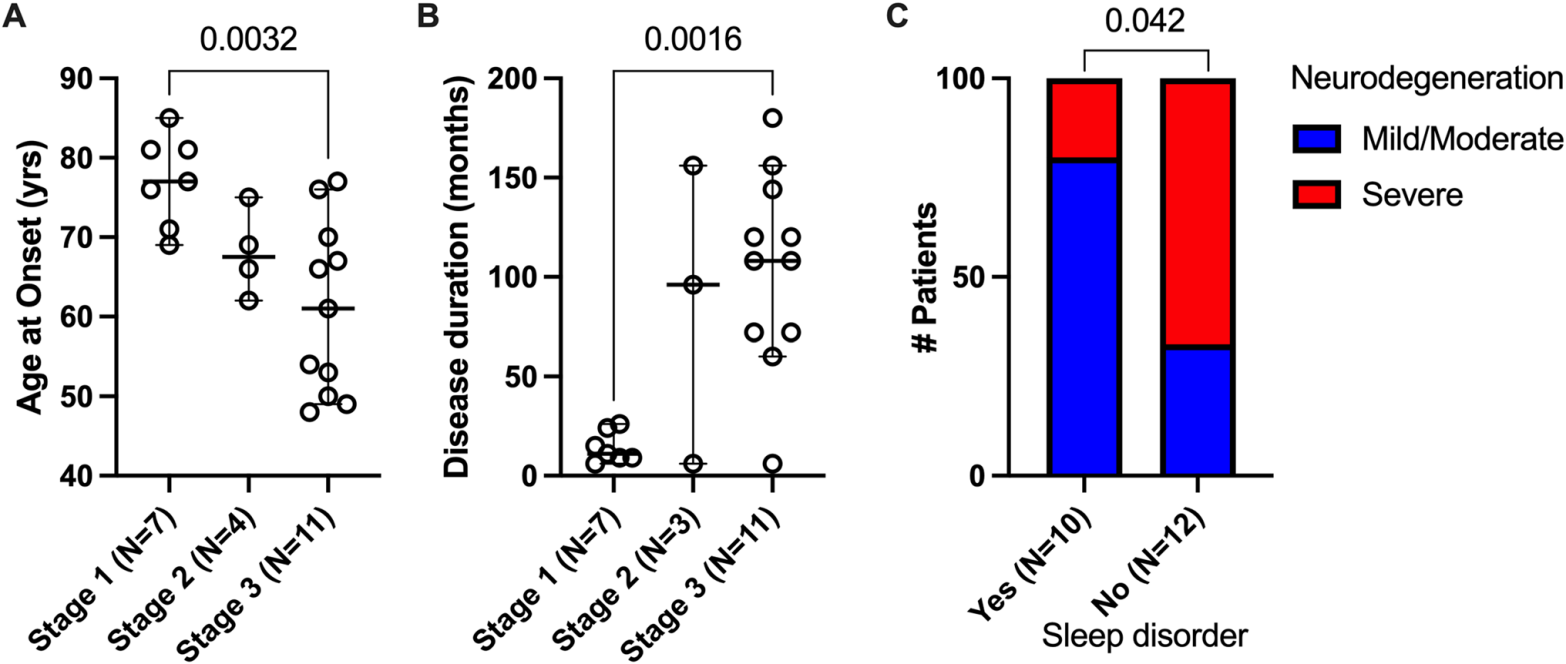
Correlation analysis between the different pathology stages and **A** age of onset and **B** disease duration; and **C** between the presence of sleep disorder and degree of neurodegeneration (**A**, **B** one-way ANOVA with Tukey’s multiple comparisons test, **C** Fisher’s exact test)

**Fig. 6:**
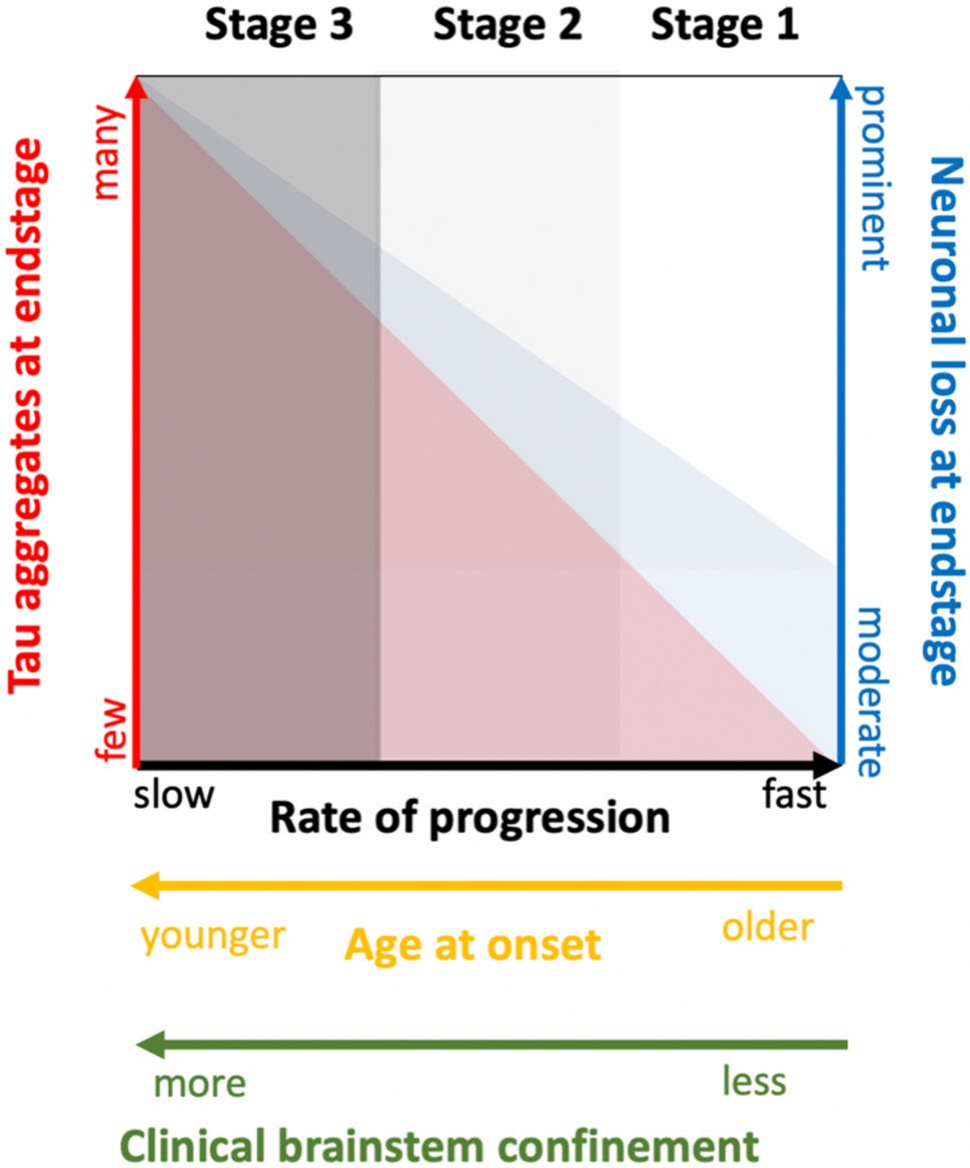
Potential relationship between grades of severity of neurodegeneration of the brainstem (mild—severe), tau accumulation (few—prominent), age at onset (older—younger) and rate of disease progression (fast – slow)

Clinical features and their corresponding pathological stages (*stages 1 to 3*) for each patient are detailed in Tables 1 and 3. The patients with *stage 3* pathology and severe neurodegeneration were younger at disease onset (48–77 years; median 61 years) and had a longer disease duration (median 9 years) compared to patients at *stage 1* and *stage 2*, who were older (69–85 years; median 79 years) and had a shorter disease duration (median < 1 year) (Tables 1 and 3; Figs. 5, 6, suppl. Fig. 3). Clinical symptoms at onset were similar at *stage 1*,* stage 2* and *stage 3* with the exception of sleep disorder that was more common in patients with moderate/severe tauopathy (Fig. 5C). Correlations with other symptoms are shown in suppl. Fig. 5. Patients at *stage 3* developed significantly more oculomotor abnormalities during the course of the disease (Table 1, suppl. Table 2). Concerning cognition, despite the significantly longer duration of disease, only 3/11 patients at *stage 3* pathology showed at most mild cognitive impairment, whereas 4/11 patients at *stage 1 or 2* developed pronounced cognitive impairment during the course of the disease. However, these patients had slightly more PART-like tau pathology in the hippocampus/limbic system (neurofibrillary stage III) than the younger patients in *stage 3* (neurofibrillary stage II) and more widespread ßA4 amyloid deposits.

**Table 3:** Summary of demographic and clinical features in patients of different disease stages of anti-IgLON5 pathology

	*Stage 1* (*n* = 7)	*Stage 2* (*n* = 4)	*Stage 3* (*n* = 11)
Age at onset, median (range)	79 (69–85)	66 (62–66)	61 (48–77)
Age at death, median (range)	79.5 (77–86)	75 (72–76)	70 (59–87)
M/F	5/1	3/0	5/6
Subacute *vs* chronic clinical course	7/1	2/2	1/10
Disease course (in months) median (range)	10 (6–24)	96 (66–156)	108 (6–180)
Comorbidities*—yes/no	2/5	1/3	1/10
Phenotype at onset^#^ (*n*)	Sleep disorder (5), bulbar syndrome (3), movement disorder (1)	PSP-like (3), MN (1)	Bulbar syndrome (7), sleep disorder (3), PSP-like (2), movement disorder (1)
CSF pleocytosis—yes/no/unknown	3/3/1	0/3/1	1/4/6
HLA-DRB1*10:01—yes/no/not tested	4/0/2	1/1/1	4/3/4
Immunotherapy—yes/no	6/1	2/2	5/6
Response to immunotherapy—yes/no	3/3	1/1	2/3

*Cancer or systemic autoimmune diseases

^#^More than one possible

Consistent with the anatomical distribution of the neuropathological findings with prominent involvement of the anterior horns of the spinal cord, the patient at *“stage 1 and brainstem* ± *spinal TDP-43 pathology*” (patient #6, Tables 1, 2) and the patient with “*stage 2 without TDP-43 pathology*” (patient #11) showed, in addition to bulbar dysfunction, the clinical picture of motor neuron disease with marked muscle atrophy, paresis, and fasciculations. These symptoms were not clinically evident in the other patients with brainstem ± spinal TDP-43 co-pathology associated with tau lesions at *stage 3*. A higher female to male ratio was observed in the TDP-43 positive cases (4:2) compared to the TDP-43 negative group (3:13). Beyond this, the patient groups did not differ in terms of their age at onset (median 69.5 years [TDP-43] vs. 67 years [non-TDP-43]) or the distribution of initial clinical symptoms.

The risk HLA haplotype HLA-DQB1*05:01 was equally represented in cases with mild/moderate and severe neurodegeneration, whereas the HLA-DRB1*10:01 allele was more frequently represented in cases with mild/moderate neurodegeneration (suppl. Fig. 5C). No statistical significance was reached when comparing its distribution between stages, age at onset or the presence of a PSP phenotype (suppl. Fig. 4). The limited availability of other laboratory parameters like CSF pleocytosis and anti-IgLON5 antibody titers in CSF and/or serum prevented us to draw firm conclusions (suppl. Fig. 5C).

### Applicability of the original neuropathological criteria of the anti-IgLON5 disease-related tauopathy and proposed update

Of the 22 cases, 15 (68%) fulfilled the original neuropathological research criteria of a brainstem-predominant tauopathy (11 definite, 4 probable). In 12/15 (80%), the tau pathology consisted of a mixture of 3R and 4R tau isoforms, whereas in the other three cases, 4R tau isoforms predominated. In contrast, 7/22 cases (32%) did not meet the neuropathological criteria of the anti-IgLON5 disease-related tauopathy due to the lack of overt/prominent brainstem tau pathology (Fig. 4), although patients had the clinical features of anti-IgLON5 disease and had anti-IgLON5 antibodies.

In view of these findings and the spectrum of pathologies observed in anti-IgLON5 disease in an extended autopsy series, and keeping in mind that the disease as such is defined by (1) the presence of anti-IgLON5 antibodies in the CSF and/or serum, and (2) the presence of neurological symptoms reflecting a predominantly brainstem involvement, we propose adapting the original research criteria that defined the unique tauopathy associated with the disease [23] accordingly (Table 4), and to delete the diagnostic category “possible”.

**Table 4:** Updated neuropathological criteria to define the tauopathy associated with anti-IgLON5 disease

*A: Probable*
Neuropathological findings of a brainstem-predominant tauopathy including all of the following requirements:
1. Neurodegenerative features with neuronal loss and gliosis in brain areas showing hyperphosphorylated (p)Tau pathology
2. Selective neuronal involvement by deposition of pTau in the form NFT, pretangles and neuropil threads with both 3R tau and 4R tau, or rarely 4R tau predominant isoforms contributing to the inclusions^a^
3. The burden of pTau pathology is moderate (*stage 2*) or severe (*stage 3*) and predominantly affects subcortical structures, including the hypothalamus, brainstem tegmentum and/or upper spinal cord^b^
AND
one of the following **supportive features**:
1. Clinical history suggestive of anti-IgLON5 disease (sleep disorder (NREM and REM parasomnia with sleep apnea), bulbar symptoms, gait instability and chorea or facial dyskinesias)
2. Presence of HLA-DRB1*1001 and HLA-DQB1*0501 alleles
AND
Reasonable exclusion of an alternative diagnosis
*B: Definite*
Criteria for “probable”
AND
Presence of IgLON5 antibodies in CSF and/or serum
OR
Neuropathological findings show only a minimal tau pathology (as defined in *stage 1*, see text) that does not fulfill all requirements for “probable”, but shows neurodegenerative features with variable neuronal loss and gliosis in the hypothalamus and/or brainstem and/or spinal cord
AND
Presence of anti-IgLON5 antibodies in CSF or serum
AND
one of the following **supportive features**:
1. Clinical history suggestive of anti-IgLON5 disease
2. Presence of HLA-DRB1*1001 and HLA-DQB1*0501 alleles

^a^Scattered oligodendroglial inclusions and ramified astrocytes may be observed

^b^Hippocampus generally involved

As it is still unclear whether the presence of a 4R tau predominant tauopathy is a separate subtype or an earlier/later stage of the disease, we recommend that the diagnosis should be only established in the presence of anti-IgLON5 antibodies in the CSF and the presence of brainstem symptoms including a sleep disorder and/or a supportive HLA genetic background. The same holds for cases where neuropathological findings are those of *stage 1*, with no overt or only mild tau pathology in the brainstem, which may also be identified in individuals without symptoms of a neurodegenerative process [9, 10]. In this situation, as well as in cases with TDP-43 proteinopathy “only”, we also recommend establishing the diagnosis in an integrated manner, i.e., in the presence of anti-IgLON5 antibodies in the CSF and the presence of brainstem symptoms including a sleep disorder and/or a supportive HLA genetic background. Conversely, as autoantibodies at certain titers may be rarely discovered as an incidental finding of a panel workup of individuals without clinical features consistent with the disease, as well as the possibility of false-positive autoantibody testing that occurs in any clinical testing scheme [2], the diagnosis of anti-IgLON5 disease should be only established in the presence of CSF antibodies and clinical symptoms.

Due to the consistent involvement of the brainstem and the hypothalamus as observed in the pathology burden map, we suggest that the neuropathological assessment could be performed in a minimal set of brain regions that represent the “high interest areas” of the disease. These areas partly overlap with other samplings protocols, e.g., as suggested for PSP [47] and should be easily applicable by other investigators. They should include at least the perirolandic region, striatum and/or lenticular nucleus, hypothalamus, hippocampus, midbrain, pons, medulla oblongata, and cerebellum. If a broad sampling is locally possible, it should include further brain regions (see “Materials and methods”), and the spinal cord.

## Discussion

We present new neuropathological observations in a larger autopsy series of 22 patients affected by anti-IgLON5 disease and propose adaptation the original neuropathological research criteria of its unique tauopathy based on the new findings.

First, we observed a spectrum of the burden of tau pathology and associated neurodegenerative features in the core brain regions affected by the disease. We suggest to integrate them into three different *stages* that imply a progression of the pathology: one end of the spectrum, *stage 3*, is characterized by preferentially hypothalamic and brainstem degeneration and is associated with extensive phosphorylated tau pathology, as originally described [23, 49]. *Stage 2* represents an intermediate stage, with mild-to-moderate brainstem degenerative features and with mild-to-moderate tau pathology involving a subset of the neuroanatomical regions affected at *stage 3*. The other end of the spectrum, *stage 1*, is also characterized by brainstem degeneration, usually in a mild(er) form, and has no obvious or only minimal thread p-tau pathology as assessed by conventional AT8 immunohistochemistry (32% of cases).

Cases at *stage 1,* however, could not be well classified based on the 2016 neuropathological criteria due to the lack of overt tau pathology. These cases are particularly difficult if not impossible to identify without the knowledge of the clinical symptoms and the presence of anti-IgLON5 antibodies, as similar changes may be also observed with aging. The application of special immunostainings that reflect neuronal alterations, astrogliosis and/or microglial activation may be helpful for the identification of the neurodegenerative process in the brainstem and/or hypothalamus. However, microglial activation and the formation of microglial nodules in the lower brainstem are a relatively frequent finding in the terminal state of severe illnesses of different types, although they involve more frequently the dorsal nucleus of the vagal nerve, the trigeminal nuclei or the inferior olives, though not exclusively. In contrast, immunostaining for IgG4 in the brainstem, particularly the medulla oblongata and cerebellum, may provide a diagnostic clue pointing towards anti-IgLON5 disease, as recently suggested [8], but will always require the determination of the antibody status in the CSF and/or serum for establishing the diagnosis. Immunostaining for IgG1 has been shown to be negligible [8], while IgG2 and IgG3 are usually underrepresented in the CSF of patients with anti-IgLON5 disease [50]. Other more specific disease markers for the assessment of early neuropathology stages of anti-IgLON5 disease are urgently needed. Identification of markers of earliest tau pathology, before phosphorylation at classical phospho-sites occur, or other more specific markers related to the presence of anti-IgLON5 antibodies may help to identify these cases with short(er) disease duration.

While a progressive accumulation of tau over time might be expected from what we have learned about misfolded proteins over the last decades, the shorter disease duration in older patients at *stage 1* is intriguing. Whether a reduced resilience or higher risk of mortality in advanced age accounts for the shorter disease duration, or different disease mechanism are involved at different ages, is currently unclear. The presence of concomitant pathologies, as those frequently observed at this age group in individuals without obvious neurological symptoms (“age-related” pathologies) [31, 44] might have contributed to some of the neurological symptoms, including cognitive dysfunction. Although the overall co-pathology load was too low to justify the rapid deterioration of the patients or the brainstem dysfunction, their existence may contribute to a reduced ‘brain reserve’ in the elderly and reduce the tolerance to the functional effects of the anti-IgLON5 antibodies. Recently, a dosage-dependent relationship between the “at risk” HLA haplotype and the age at onset has been found, reflected by increasing age of disease onset in carriers of the less “at-risk” genotype, and an overall lower risk of disease in subjects carrying the DQ1 alleles [57]. The number of cases in our study not carrying the at-risk haplotype was too low to find an association, although we observed that the HLA-DRB1*10:01 allele was more frequently represented in cases with mild/moderate neurodegeneration while the HLA-DQB1*05:01 allele was equally represented in cases with mild/moderate and severe neurodegeneration. Moreover, CSF pleocytosis was more frequently observed in cases with mild/moderate than with severe brainstem neurodegeneration. More studies will be required to confirm these findings also in larger neuropathological series.

Whether the differences in the tau burden represent indeed different “stages”, which imply a progression of the pathology, or are “patterns” (low tau load *vs* moderate/high tau load) at specific time points, is still unclear, although detailed neuroanatomical mapping and experimental data support the first. If *stage 1* indeed represents an early disease stage from a neuropathological point of view and *stage 3* a more advanced one, it reinforces the concept that tau accumulation is a secondary, time-dependent phenomenon related to the presence of anti-IgLON5 antibodies.

While the tau pathology at *stage 3* had a similar distribution of 3R and 4R isoforms in the brainstem in most cases, three patients had a 4R tau predominant pathology. In these three cases, however, the distribution of pathology was similar to the other *stage 3* cases and was predominantly neuronal. One of these patients had in addition a severe nigral degeneration and presented clinically with a PSP phenotype. Another patient with a long disease duration who had been retrospectively identified, had neuropathological features that were formally consistent with a diagnosis of PSP, including neuronal and glial tau pathology with some, but not many, typical tufted astrocytes in the basal ganglia, but a predominant and severe brainstem involvement [8, 47], suggesting a brainstem variant of PSP. This patient had overlapping clinical symptoms of PSP and also of anti-IgLON5 disease (for clinical details see: [8]), and had anti-IgLON5 antibodies in CSF. This case is intriguing, and it is currently very difficult to discern whether a PSP-type pathology masks, mimics or is indeed part of the expectable spectrum of the anti-IgLON5 disease-related pathology. It is, however, difficult to understand why a minority of patients show a 4R tau isoform predominant pathology, as cryoEM studies are convincingly showing a very strong association of the tau filament molecular structure (or its “folding signature”) with a specific disease, which would be expected also for anti-IgLON5 disease [41, 43]. Further studies in this direction are definitely required to deepen our understanding of the particular biological properties of the tau filaments in this immunological context, and the effects of potential genetic modifiers or susceptibility factors for a particular tauopathy phenotype, as well as the effect of immunomodulatory and neuroprotective therapies on this condition.

Whether the distribution of pathology around the 3rd and 4th ventricle along the midline relates to the different constitution of the blood–brain barrier or the immediate contact of the CSF along the ependymal lining, representing a potential gate for the anti-IgLON5 antibodies to boost into the CNS is still a matter of speculation. The symptoms are likely related to prominent alteration of neuronal function in the different brainstem areas, particularly in the pons and medulla, and correlated more with the neuroinflammatory and neurodegenerative process per se than with the presence or amount of tau pathology. Single in vivo neuroimaging studies in this direction have been recently performed [56]. These findings also support the notion that the accumulation of misfolded tau in the CNS likely represents a delayed, secondary phenomenon, as it also occurs in subacute sclerosing panencephalitis (SSPE) [6], postencephalitic parkinsonism (PEP) [11], chronic traumatic encephalopathy (CTE) [37], and likely in Guam PDC (Parkinson-dementia complex) and the Nodding syndrome [42]. This aspect is particularly supported by the mild/moderate but detectable brainstem p-tau pathology observed in the intermediate *stage 2* cases, and the presence of isolated threads in the clue regions in some of the patients at *stage 1*, all of which had anti-IgLON5 antibodies in CSF and typical symptoms.

Second, we have observed concomitant TDP-43 protein aggregates (neuronal and fewer oligodendroglial) in about one-third of cases with anti-IgLON5 disease, associated or not with the extensive tauopathy. These inclusions particularly affected the neurons of the brainstem tegmentum, as well as neurons of the anterior horns of the spinal cord, areas that were expected to contain tau pathology [23], but also overlap with those affected in MND. While oculomotor dysfunction is rare in ALS, ophthalmoparesis and alteration of pursuit, nystagmus and saccades have been described in patients with long disease duration [52]. In neuropathological studies, fine filamentous TDP-43 accumulations in oculomotor neurons have been identified in nearly 50% of ALS cases [39] but also in about 16% of non-ALS elderly subjects over 80 years with associated AD-neurodegenerative changes [39]. On the other hand, two patients with anti-IgLON5 disease-related tauopathy at *stage 3* (extensive tau pathology) and one at *stage 2* without TDP-43 pathology, had also motor neuron symptoms as well as tau pathology and/or neuronal loss in the spinal cord and hypoglossal nucleus, supporting the notion that the MN vulnerability is also part of anti-IgLON5 disease, independently of the accumulation of TDP-43 protein.

In most of the cases, the TDP-43 pathology was associated with the classical extensive brainstem tauopathy observed in *stage 3.* In one patient (patient # 6) the TDP-43 pathology was the main one (*stage 1* + TDP-43). Such cases, where TDP-43 aggregates are detectable in the absence of or with only minimal tau pathology, challenge the diagnosis of anti-IgLON5 disease by neuropathology alone, as there are no obvious distinctive features that could point to anti-IgLON5 disease. They require the presence of anti-IgLON5 antibodies in CSF and neurological symptoms of at least a brainstem dysfunction for establishing a diagnosis. Moreover, no criteria can be currently applied to make anti-IgLON5 disease-related pathology *stage 1* with minimal tau (or tau negative) the “primary phenotype” and the TDP-43 associated motor neuron pathology the “secondary phenotype”. A similar difficulty may arise in cases with limbic TDP-43 proteinopathy (LATE), although we observed this pathology only in a single case with the characteristic brainstem tau pathology.

The presence of neuronal TDP-43 pathology in the subthalamic nucleus, globus pallidus and substantia nigra in a single case, regions that are known to be also involved in PSP and corticobasal degeneration (CBD) tauopathies [4], could also be reminiscent of the distribution of the pallido-luysian phenotype of ALS, as described particularly in Asia. A pallido-nigro-luysian atrophy associated with TDP-43 is also characteristic of Perry syndrome, an autosomal dominant parkinsonism with central hypoventilation [38], caused by mutations in the dynactin subunit 1 gene (*DCTN1*). While clinical features may overlap with those of anti-IgLON5 disease, the neurodegenerative features with prominent neuronal loss, axonal spheroids and pigment that are characteristic findings in Perry syndrome were absent in all cases with anti-IgLON5 disease so far. However, we are not aware of any studies analyzing mutations in the *DCTN1* gene in anti-IgLON5 disease cases or, conversely, the presence of anti-IgLON5 antibodies in Perry syndrome. Still, patients with anti-IgLON5 disease have no family history of a neurological disorder and are considered to suffer from a sporadic disease form. Only one patient of our study with anti-IgLON5 antibodies and a PSP phenotype had been tested and did not have a mutation in *DCTN1.* More studies in this direction may be of interest.

The co-accumulation of tau and TDP-43 proteins is increasingly recognized in some tauopathies, like chronic traumatic encephalopathy or SSPE (also considered a “secondary” tauopathies) [1, 36, 41], or some primary astrogliopathies [16, 24, 32]. Recently, TDP-43 has also been found in motor neurons of the spinal cord in a series of “primary” tauopathies in 38% of PSP cases and 58% of CBD cases, but not in globular glial tauopathies (GGT) or Alzheimer’s disease [46]. The authors suggest that these two tauopathies may have some mechanistic links with the MND form of ALS-TDP. In anti-IgLON5 disease, the TDP-43 protein accumulation seems to involve primarily susceptible neurons in the areas affected by neurodegeneration and may add to the clinical phenotype. It may be postulated that the pro-inflammatory microenvironment induced by anti-IgLON5 antibodies makes neuronal populations selectively vulnerable for a particular protein pathology, leading to the accumulation of TDP-43 in motor neurons of the affected brainstem and spinal cord, as would, e.g., alpha-synuclein/Lewy bodies accumulate in pigmented and/or other vulnerable neurons of the brainstem if these regions were predominantly affected in anti-IgLON5 disease.

The limited number of TDP-43-positive cases in our study prevented us to predict a particular pattern or stages of this pathology. A systematic analysis of more autopsy cases and experimental studies will be needed to clarify whether the presence of TDP-43 aggregates is the result of a synergistic or downstream effect of tau accumulation or depends on some genetic modifier, inflammatory microenvironment, disruption of the blood–brain barrier, patient’s age, disease duration, or therapeutic effect, and/or represents a particular subtype of anti-IgLON5 disease, or is to be considered as purely incidental [54].

As these new observations introduce caveats and challenges for the neuropathological diagnosis, we propose adapting the neuropathological criteria of the anti-IgLON5 disease-related tauopathy that were originally based on 6 autopsy cases. We also suggest a minimal set of brain regions representing the “high interest areas” of the disease, which should be easily applicable by other investigators and ensure consistency for reproducing our findings and for further studies. Importantly, the neuropathological criteria are not meant to be used for diagnosing anti-IgLON5 disease as such, as this is currently based on a very reliable immunological test capable of detecting the antibody in the CSF or serum. They are relevant for defining the unique tauopathy associated with anti-IgLON5 disease—a hallmark of this disease—, and its identification should serve as a confirmatory and/or alternative diagnostic option in cases where a bonafide in-vivo diagnosis is lacking, and for research purposes. The inclusion of clinical features and typical HLA haplotype (absent 30–40% of the patients [57], are at present only supportive features that are useful to increase the diagnostic certainty of anti-IgLON5 disease. This differs from other neurodegenerative diseases, where the definite diagnosis relies on neuropathological findings and not on a CSF/blood-based test, and reflects one of the unique features of anti-IgLON5 disease, the combination of autoimmunity and neurodegeneration.

In conclusion, a higher number of autopsies has led to the observation of a broader spectrum of anti-IgLON5 disease-associated neuropathologies that can be currently stratified in a continuum of progressive brainstem neurodegeneration and tau accumulation that ranges from *stage 1*, an early “minimal/non-tau brainstem degeneration”, to *stage 3*, the extensive anti-IgLON5 disease-related “brainstem-predominant 3R + 4R tauopathy”, with some variations, and with or without other concomitant protein aggregates, particularly TDP-43.

The common denominator of this spectrum is the same topographic distribution of the neurodegenerative process that involves midline structures, i.e., the hypothalamus, brainstem and spinal cord, which explained most of the clinical symptoms.

These observations support the concept that the accumulation of tau filaments is indeed a secondary, likely time-dependent phenomenon, as observed, e.g., in postencephalitic parkinsonism, subacute sclerosing panencephalitis, or chronic traumatic encephalopathy [43].

The proposal of different stages intends to reflect the possible spectrum and/or potential subtypes of brain pathologies underlying different clinical phenotypes. Nevertheless, there are still important unanswered questions that require additional experimental data and assessment of more cases, and can not yet be resolved with current data. Furthermore, as with many staging systems and classifications, it has to be considered a human construct to better understand and analyze disease profiles from different perspectives, and as such, it should be considered a dynamic one that, despite our efforts to include accurate information, it may be subject to change based on new observations. It might be also important to widen the screening for anti-IgLON5 antibodies to other neurological symptoms that may overlap with other more frequent neurodegenerative conditions, such as, e.g., PSP or ALS, particularly when these are associated with atypical symptoms [54] and likely also to a subset of TDP-43 proteinopathies.

## Supplementary Information

Below is the link to the electronic supplementary material.

**Supplementary Fig. 1: Particular tau immunoreactivity patterns**. Particular types of tau immunoreactivity that have been observed in the anti-IgLON5 disease-related tauopathy. **a**: tau positive threads in the infundibulum, tau positive threads in subependymal areas, **c,d**: focal tau immunoreactivity in the glomerula of the cerebellum with single positive Cajal cells (inset). Purkinje cells remain usually negative (TIF 7484 KB)

**Supplementary Fig. 2: Motor neuron pathology in the anterior horn of the spinal cord.** **a** Marked neuronal loss in the anterior horn of the spinal cord. **b**, **c** Residual neurons appear chromatolytic. **d** There are also some axonal spheroids, here adjacent to the perikaryon of the motor neuron (arrow). **e** Single neuronophagias can be also observed (arrow). In addition, small eosinophilic inclusions consistent with Bunina bodies can be identified in single neurons. **g**–**i** The affected areas of the anterior horns may show variable density of tau-positive threads, which are usually also detected in the posterior horns (inset in **i**). **j**–**l** pTDP-43 pathology can be also observed in some cases in association with or independently of the presence of tau pathology. The immunoreactivity follows mostly a diffuse-granular cytoplasmic pattern (**j**, **k** lower panel), but skein-like inclusions (**k**, upper panel), can also be detected. In addition, small colied-body like oligodendroglial inclusions can be present (**l**) (TIF 18167 KB)

**Supplementary Fig. 3:** Correlation analysis between the different pathology stages and selected clinical parameters (**A**–**B**), disease duration and age of onset (**C**) and between antibody titers and disease duration (**D** limited availability of data, *n = 7*) (**A**, **B** Fisher’s exact test, **C** unpaired two-tailed t-test, **D** linear regression analysis using the Pearson correlation coefficient) (TIFF 432 KB)

**Supplementary Fig. 4:** Correlation analysis between different “risk” HLA haplotypes, pathology stages and clinical parameters (Fisher’s exact test) (TIFF 238 KB)

**Supplementary Fig. 5:** Correlation analysis between neurodegeneration and selected clinical parameters (**A**–**B**), and between neurodegeneration and selected laboratory parameters (**C** antibody titers: limited availability of data, *n* = 7) (**A**, **B** Fisher's exact test, **C** unpaired two-tailed t-test and Fisher’s exact test) (TIFF 961 KB)

**Supplementary Fig. 6:** Frequencies of combinations of Tau and TDP-43 pathologies across the different disease stages (**A** one-way ANOVA with Tukey's multiple comparison's test, **B** Fisher's exact test) (TIFF 334 KB)

**Supplementary Table 1:** Suggested stages of pathology and original criteria for “possible”, “probable” and “definite” categories to define the tauopathy underlying the anti-IgLON5 disease as originally proposed in 2016 (DOCX 16 KB)

**Supplementary Table 2:** Details of clinical symptoms at onset and during disease evolution (DOCX 26 KB)

**Supplementary Table 3:** Details of the immunohistochemical stainings that have been performed in the different brain areas for the analysed cases. Numbers represent the case number in Tables 1 and 2 (DOCX 15 KB)

## Data Availability

All data supporting the findings of this study are available within the paper and its supplementary information.
