# Supplementary figures and images for: Neuropathological spectrum of anti-IgLON5 disease and stages of brainstem tau pathology: updated neuropathological research criteria of the disease-related tauopathy
Source: Acta Neuropathol. 2024 Oct 14;148(1):53. doi: 10.1007/s00401-024-02805-y (PMC11473580; doi:10.1007/s00401-024-02805-y)

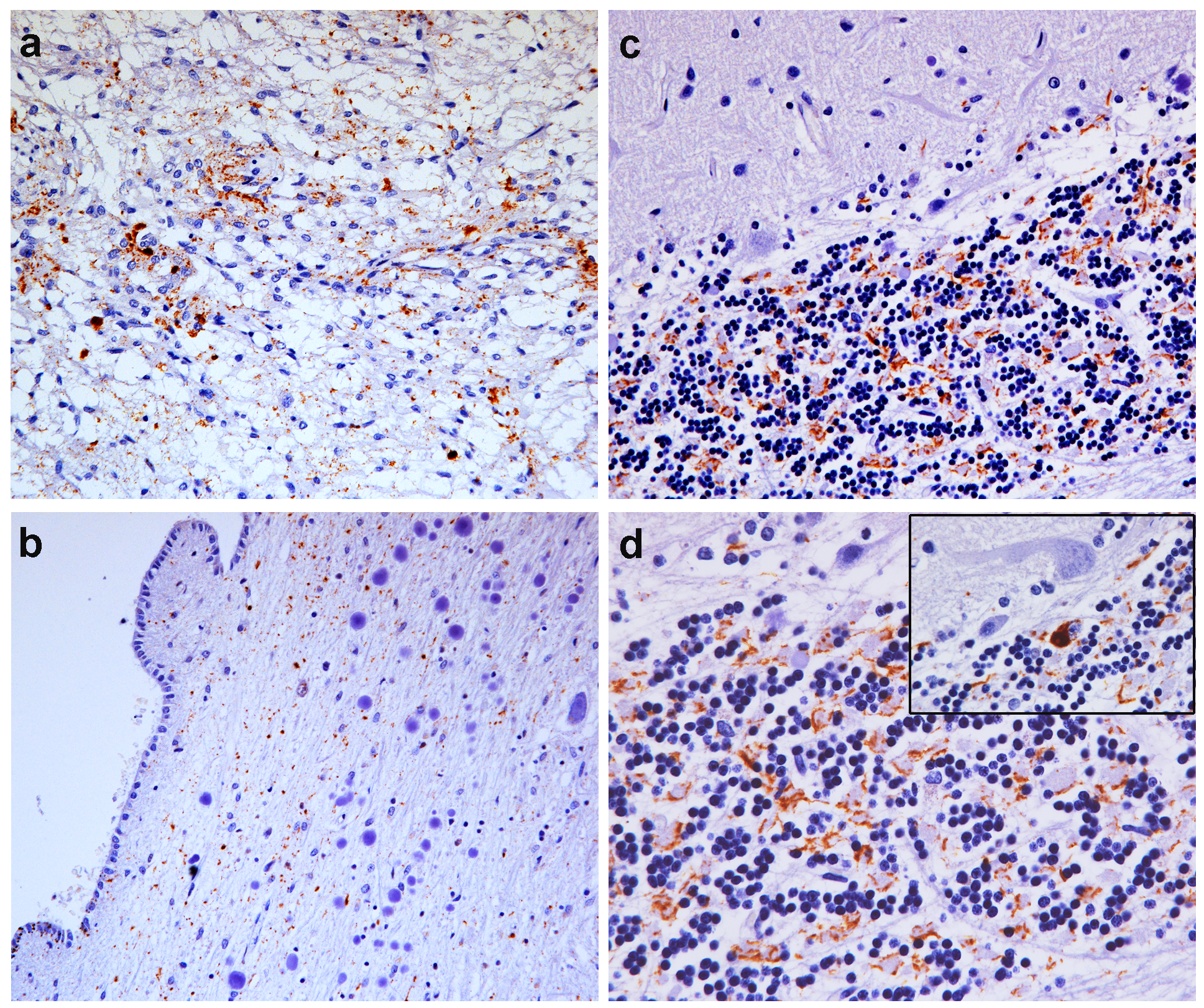

Supplement: Supplementary file 1 — Supplementary Fig. 1: Particular tau immunoreactivity patterns. Particular types of tau immunoreactivity that have been observed in the anti-IgLON5 disease-related tauopathy. a: tau positive threads in the infundibulum, tau positive threads in subependymal areas, c,d: focal tau immunoreactivity in the glomerula of the cerebellum with single positive Cajal cells (inset). Purkinje cells remain usually negative (TIF 7484 KB) [file 401_2024_2805_MOESM1_ESM.tif]

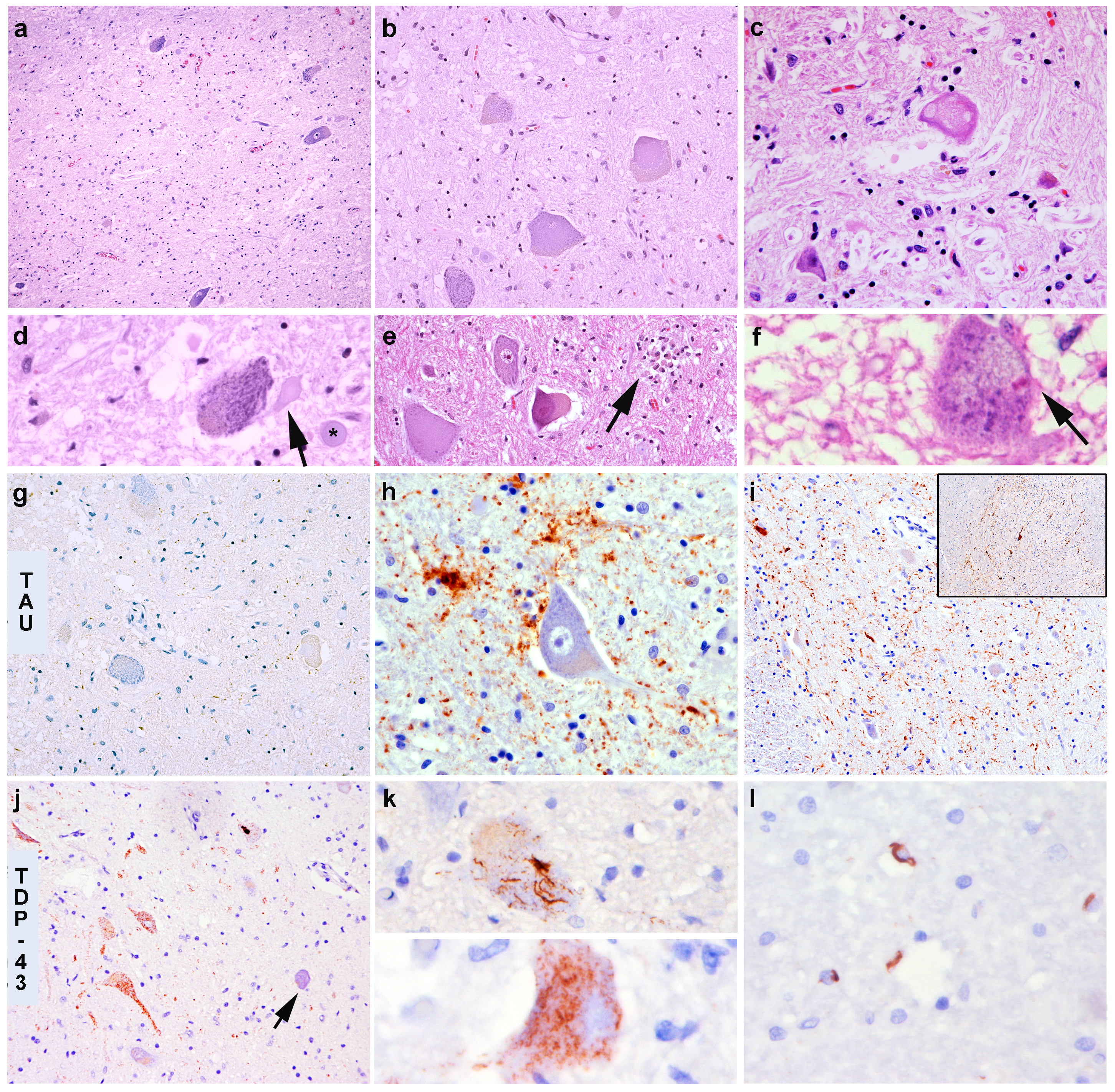

Supplement: Supplementary file 2 — Supplementary Fig. 2: Motor neuron pathology in the anterior horn of the spinal cord. a Marked neuronal loss in the anterior horn of the spinal cord. b, c Residual neurons appear chromatolytic. d There are also some axonal spheroids, here adjacent to the perikaryon of the motor neuron (arrow). e Single neuronophagias can be also observed (arrow). In addition, small eosinophilic inclusions consistent with Bunina bodies can be identified in single neurons. g–i The affected areas of the anterior horns may show variable density of tau-positive threads, which are usually also detected in the posterior horns (inset in i). j–l pTDP-43 pathology can be also observed in some cases in association with or independently of the presence of tau pathology. The immunoreactivity follows mostly a diffuse-granular cytoplasmic pattern (j, k lower panel), but skein-like inclusions (k, upper panel), can also be detected. In addition, small colied-body like oligodendroglial inclusions can be present (l) (TIF 18167 KB) [file 401_2024_2805_MOESM2_ESM.tif]

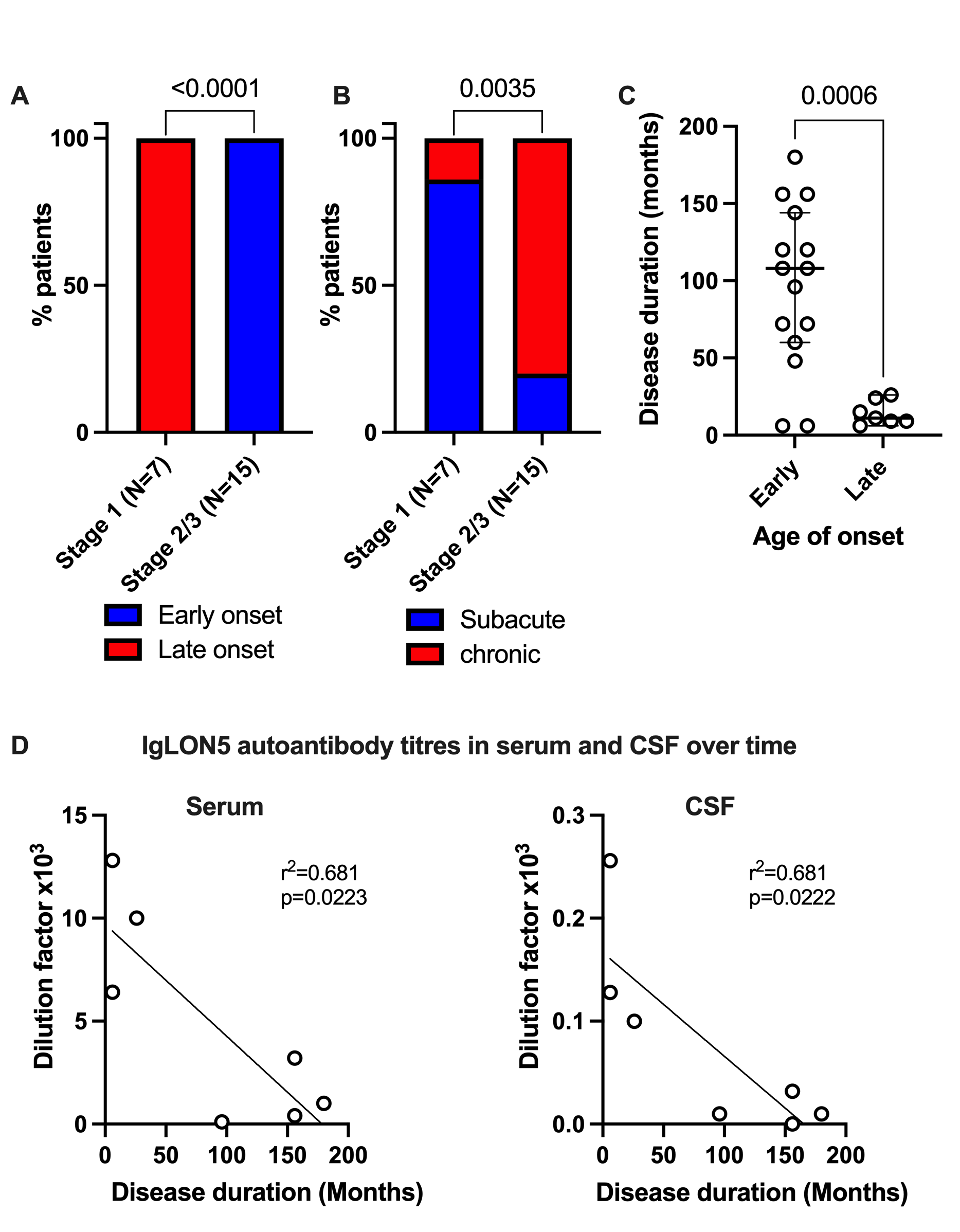

Supplement: Supplementary file 3 — Supplementary Fig. 3: Correlation analysis between the different pathology stages and selected clinical parameters (A–B), disease duration and age of onset (C) and between antibody titers and disease duration (D limited availability of data, n = 7) (A, B Fisher’s exact test, C unpaired two-tailed t-test, D linear regression analysis using the Pearson correlation coefficient) (TIFF 432 KB) [file 401_2024_2805_MOESM3_ESM.tiff]

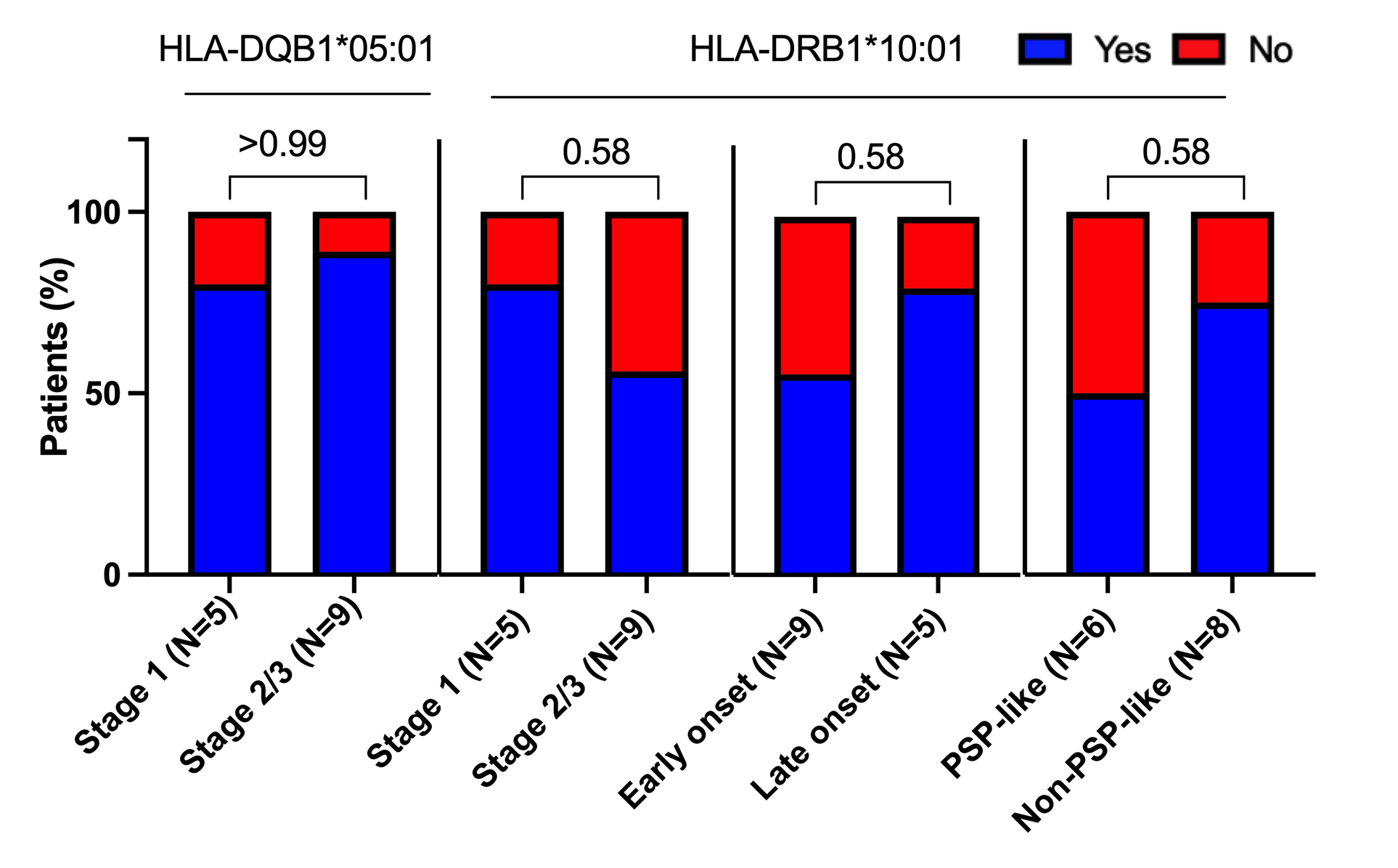

Supplement: Supplementary file 4 — Supplementary Fig. 4: Correlation analysis between different “risk” HLA haplotypes, pathology stages and clinical parameters (Fisher’s exact test) (TIFF 238 KB) [file 401_2024_2805_MOESM4_ESM.tiff]

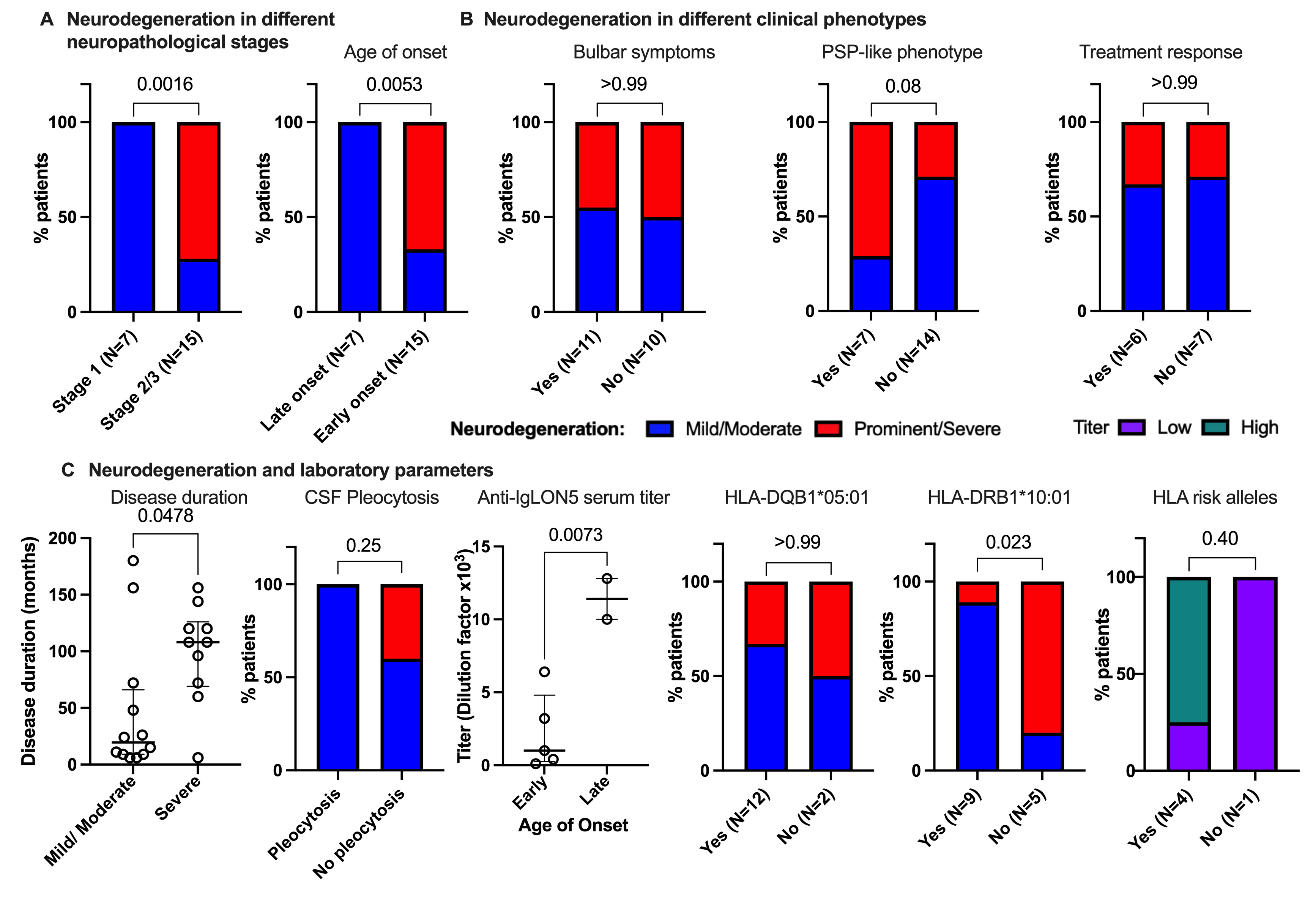

Supplement: Supplementary file 5 — Supplementary Fig. 5: Correlation analysis between neurodegeneration and selected clinical parameters (A–B), and between neurodegeneration and selected laboratory parameters (C antibody titers: limited availability of data, n = 7) (A, B Fisher's exact test, C unpaired two-tailed t-test and Fisher’s exact test) (TIFF 961 KB) [file 401_2024_2805_MOESM5_ESM.tiff]

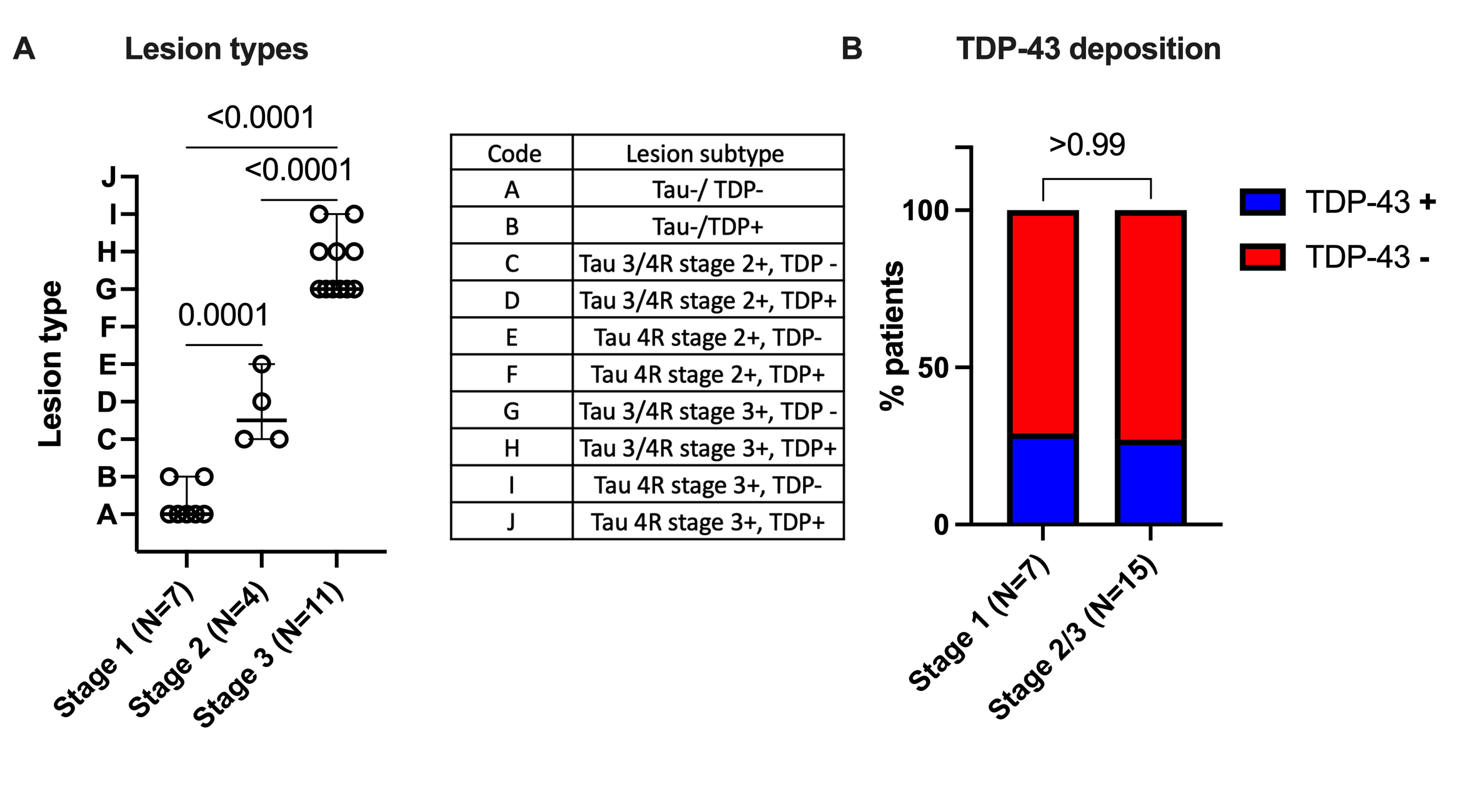

Supplement: Supplementary file 6 — Supplementary Fig. 6: Frequencies of combinations of Tau and TDP-43 pathologies across the different disease stages (A one-way ANOVA with Tukey's multiple comparison's test, B Fisher's exact test) (TIFF 334 KB) [file 401_2024_2805_MOESM6_ESM.tiff]
