# Supplementary material for: Neuropathological spectrum of anti-IgLON5 disease and stages of brainstem tau pathology: updated neuropathological research criteria of the disease-related tauopathy
Source: Acta Neuropathol. 2024 Oct 14;148(1):53. doi: 10.1007/s00401-024-02805-y (PMC11473580; doi:10.1007/s00401-024-02805-y)
Supplement: Supplementary file 7 — Supplementary Table 1: Suggested stages of pathology and original criteria for “possible”, “probable” and “definite” categories to define the tauopathy underlying the anti-IgLON5 disease as originally proposed in 2016 (DOCX 16 KB) [file 401_2024_2805_MOESM7_ESM.docx]

**Supplementary Table 1: Original criteria**

Depending on clinical features and antibody status, cases can be classified as “definite”, “probable” or “possible” cases, as suggested in the original research criteria [23], and summarized below:

| **Possible** |
| --- |
| All of the following requirements |
| Neurodegenerative features with neuronal loss and gliosis in brain areas showing hyperphosphorylated (p)Tau pathology without the presence of inflammatory infiltrates |
| Selective neuronal involvement by deposition of pTau in the form NFT, pretangles and neuropil threads with both 3R-tau and 4R-tau isoforms contributing to the inclusions |
| The pTau pathology predominantly affects subcortical structures^a^, including the hypothalamus, brainstem tegmentum and upper spinal cord |
| **Probable** |
| Criteria of “possible” AND at least one of the following |
| Clinical history suggestive of a sleep disorder (NREM and REM parasomnia with sleep apnea), or brainstem, mainly bulbar dysfunction^b^ |
| Presence of HLA-DRB1*1001 and HLA-DQB1*0501 alleles |
| **Definite** |
| Criteria for “possible” AND presence of IgLON5 antibodies in CSF or serum^c^ |

1. ^a^Hippocampus generally involved, except for one patient
2. ^b^Includes dysarthria, dysphagia, central hypoventilation, stridor
3. ^c^IgLON5 positivity is detected by cell-based assay in serum at 1/40 and in CSF at 1/2

**Additional information extracted from the original criteria**

Increasing “cranio-caudal gradient of severity from the midbrain to medulla oblongata, reaching the upper cervical cord. The cerebral cortex, basal ganglia, thalamus and subthalamic nucleus were mostly unaffected or mildly affected. In five cases, the entorhinal cortex and hippocampus were also affected, including the dentate gyrus and CA4 to CA1 sectors, with variable involvement of the transentorhinal region. Interestingly, CA2 sector was consistently involved. There was a lack of glial tau pathology except for the presence of isolated coiled bodies and granular fuzzy astrocytes in the hypothalamus and amygdala in cases 3 and 4. There were no other abnormal deposits of proteins, or if they were present, they were mild and localized in a few areas, suggesting the co-existence of other neurodegenerative or age-related pathologies. Case 2 showed isolated Bunina bodies and TDP-43 protein aggregates in a few brainstem and motor neurons in the spinal cord. A few neuritic plaques were observed in cases 2, 4, and 6, along with cerebral amyloid angiopathy in cases 2 and 4. Case 4 additionally showed scattered alpha-synuclein positive Lewy bodies and Lewy neurites in the substantia nigra and dorsal nucleus of the vagus nerve and argyrophilic grain pathology, and case 5 showed sparse neuronal cytoplasmic TDP-43 protein inclusions in the granule cells of the dentate gyrus.”
