# Supplementary material for: Neuropathological spectrum of anti-IgLON5 disease and stages of brainstem tau pathology: updated neuropathological research criteria of the disease-related tauopathy
Source: Acta Neuropathol. 2024 Oct 14;148(1):53. doi: 10.1007/s00401-024-02805-y (PMC11473580; doi:10.1007/s00401-024-02805-y)
Supplement: Supplementary file 8 — Supplementary Table 2: Details of clinical symptoms at onset and during disease evolution (DOCX 26 KB) [file 401_2024_2805_MOESM8_ESM.docx]

**Supplementary Table 2**

| **Case #** | **Stage** | **Sex** | **Age at onset** | **Age at death** | **Disease duration (months)** | **Presentation** | **Clinical subtype at presentation** | **mRS at diagnosis** | **Abnormal sleep behavior** | **Sleep breathing disorder** | **Other sleep problems** | **Bulbar dysfunction** | **Gait abnormalities** | **Oculomotor abnormalities** | **Cognitive impairment** | **Dysautonomic symptoms** | **Chorea** | **Abnormal facial movements** | **Other movement disorders** | **Cerebellar symptoms** | **Lower motor-neuron symptoms** | **Other relevant symptoms** | **Immuno-Therapy** | **HLA type** |
| --- | --- | --- | --- | --- | --- | --- | --- | --- | --- | --- | --- | --- | --- | --- | --- | --- | --- | --- | --- | --- | --- | --- | --- | --- |
| 1 | 1 | male | 81 | 82 | 6 | subacute | bulbar syndrome, sleep syndrome | n/a | no | yes, central apnea | no | yes, dysarthria, dysphagia | no | no | no | no | yes, chorea of the upper limbs, with left predominance | no | no | no | no | no | IVIg w/ short time response, RTX | **DRB1*10:01, DQB1*05:01**, DRB1*15:01, DQB1*06:02 |
| 2 | 1 | male | 81 | 82 | 11 | subacute | movement disorder, cognitive impairment, epilepsy | 4 | no | no | no | no | yes, gait failure | no | yes, dementia, abulia | no | no | no | no | no | no | yes, frontal release sign (snout and palmomental reflex positive bilateral) | Steroids 3x500 mg, improvement (mainly cognitive) | n/a |
| 3 | 1 | male | 71 | 73 | 24 | chronic | sleep disorder, PSP-like syndrome | n/a | yes, complex movement automatisms, abnormal pseudo-rhythmic movements | yes, central hypoventilation | yes, insomnia | yes, dysarthria | yes, postural instability, parkinsonism | yes, palpebral ptosis | no | yes, enuresis | no | yes, velopalatine and oromandibular dyskinetic movements | yes, spontaneous myoclonus and postural tremor in upper limbs | no | no | no | IVIg, no response | **DRB1*10:01- DQB1*05:01** |
| 4 | 1 | male | 85 | 86 | 9 | subacute | sleep disorder | 3 | yes, finalistic (eating, drinking, sewing, vocalization). NREM parasomnia in PSG | yes, obstructive apnea (AHI 27) | yes, daytime sleepiness mild, no insomnia, enuresis | yes, mild dysphagia (but became severe few weeks after diagnosis), mild unilateral vocal cord palsy (no stridor), hypersalivation, no dysarthria. | yes, unsteady gait, moderate, required walker, occasional falls | no | yes, confusional episodes with hallucinations, apathy (MOCA 12) | no | no | yes, lingual dyskinesia | no | no | no | no | Steroids iv, plasmapheresis, partial and transient improvement | **DRB1*10:01- DQB1*05:01** |
| 5 | 1 + mild TDP | female | 69 | 70 | 15 | subacute | sleep disorder, cognitive impairment | n/a | yes, continuous limb movement, sleep talking | yes, central apnea | yes, daytime sleepiness mild, no insomnia, enuresis | yes, mild hoarseness | no | yes, mild vertical gaze palsy | yes, marked cognitive decline in course of disease (MMSE 16) | yes, repeated syncopes, bradycardia | no | no | yes, akathisia and subcontinuous dyskinetic movements of the limbs | no | no | yes, visual agnosia, hallucinations, delusions | IVMP, no response | DRB1*03:01/DRB1*16:01 DQB1*05:02/DQB1*02:01 |
| 6 | 1+TDP | male | 77 | 77 | 9 | subacute | bulbar symptoms, PSP-like syndrome | 4 | unknown | unknown | unknown | yes, dysarthria, difficulty opening mouth, dysphagia | yes, ataxic gait | yes, vertical gaze palsy | no | no | yes, head chorea | yes, oral dystonia | no | yes, ataxia | yes, prominent muscle atrophy, weakness and fasciculations, ALS-like motor symptoms | no | no | DRB1*08:01/**DRB1*10:01,** DQB1*04:02/**DQB1*05:01,** HLA-A*02:01/HLA-A*31:01, HLA-B*15:01/HLA-B*40:01, HLA-C*03:03/HLA-C*03:04, DQA1*01:05/DQA1*04:01, DPB1*02:01/DPB1*04:01, HLA-E*01:01/HLA-E*01:03, HLA-F*01:01/HLA-F*01:01, HLA-G*01:01/HLA-G*01:03 |
| 7 | 1 | male | 76 | 78 | 26 | subacute | bulbar syndrome, sleep syndrome | 3 | yes, sudden sleep attacks | no | no | yes, dysarthria, dysphagia | no | yes, mild vertical gaze palsy | yes, mild cognitive impairment | no | no | yes, orofacial dyskinesias | yes, progressive dyskinesia of left upper limb | no | no | no | IVMP, oral steroids, RTX | n/a |
|  |  |  |  |  |  |  |  |  |  |  |  |  |  |  |  |  |  |  |  |  |  |  |  |  |
| 8 | 2 | male | 62 | 75 | 156 | chronic | PSP-like syndrome | 3 | no | no | yes, daytime sleepiness | no | yes, unsteady gait | yes, mild bilateral ptosis | yes | no | no | no | yes, parkinsonism | yes, gait ataxia | no | no | no | DRB1*03:01/DRB1*14:01 DQB1*05:03/DQB1*02:01 |
| 9 | 2 | male | 75 | 76 | 6 | subacute | PSP-like syndrome, cognitive impairment | 3 | no | no | yes, no further information | no | yes, unsteady gait, repeated falls | yes, double vision on left gaze | yes, mild cognitive impairment | yes, recurring vertigo | no | no | no | no | no | no | IVMP, oral steroids, MMF | unknown |
| 10 | 2+TDP | male | 66 | 72 | 96 | chronic | PSP-like syndrome | 5 | yes, abnormal movements and talking | yes, central apnea | yes, excessive daytime sleepiness | yes, severe dysphagia, dysarthria | yes, postural instability | no | no | yes, syncopes, bladder incontinence, reduced heart rate variability | no | no | yes, mild bilateral rigidity | no | no | frontal lobe dysfunction (personality change, aggressive behavior) | 5x1g IVMP, oral steroid taper, RTX; improvement of consciousness | **DRB1*10:01; DQB1*05:01** |
| 11 | 2 | male | likely 69 | 73 | 48 | subacute / chronic | bulbar syndrome, ALS-like syndrome | n/a | yes | yes, central apnea | yes, insomnia, daytime sleepiness, no more details | yes, severe dysphagia, dysarthria, dysphonia; ICU required, ventilation, finally tracheostomy | no | no | confusional episodes, drowsiness, encephalopathy, possible hallucinations | yes, recurrent syncopes that required pacemaker | yes | no | myokymia, myoclonus | no | yes, hyperreflexia, EMG suggestive of MND | yes, hallucinations | no | n/a |
|  |  |  |  |  |  |  |  |  |  |  |  |  |  |  |  |  |  |  |  |  |  |  |  |  |
| 12 | 3 | male | 53 | 59 | 72 | chronic | sleep disorder | 2 | yes, abnormal movements | yes, OSAS, stridor | yes, excessive daytime sleepiness and insomnia (fragmented, non-restorative sleep) | mild dysphagia | no | no | no | enuresis, urinary urgency and hesitance | no | no | yes, akathisia | no | no | no | three cycles of intravenous steroids and cyclophosphamide | **DRB1*10:01, DQB1*05:01** |
| 13 | 3 | male | 48 | 60 | 144 | chronic | movement disorder | n/a | no | yes, change of breathing pattern | no | yes, severe dysphagia | yes, postural instability | yes, lateral and upgaze nystagmus with limitation of vertical and abduction eye movements | no | no | yes, choreiform movements in all limbs | unknown | no | yes, cerebellar dysarthria, finger-nose dysmetria, unable to tandem walk | unknown | no | no | unknown |
| 14 | 3 | male | 49 | 59 | 120 | chronic | bulbar syndrome | n/a | no | yes, OSAS | no | yes, severe dysphagia, dyspnea | no | yes, bilateral ptosis and horizontal nystagmus | no | no | no | no | no | no | atrophy of the limbs, especially triceps | no | no | unknown |
| 15 | 3 | female | 54 | 66 | 156 | chronic | sleep disorder, bulbar syndrome | n/a | yes, continuous apparently purposeful movements, vocalizations | yes, OSAS, stridor | no | yes, dysphagia, dyspnea, mild dysarthria | yes, mild broad based gait | yes, mild bilateral ptosis | no | no | no | no | no | no | no | no | no | **DRB1*10:01; DQB1*05:01** |
| 16 | 3 | female | 77 | 87 | 120 | chronic | bulbar syndrome | n/a | unknown | unknown | unknown | yes, severe dysphagia, dyspnoe, stridor, vocal cord palsy | no | no | no | no | no | no | no | no | no | no | no | unknown |
| 17 | 3 | female | 61 | 70 | 108 | chronic | PSP-like syndrome | 2 | unknown | yes, stridor and obstruction due to vocal cord paresis | yes, daytime sleepiness | yes, dysphagia, vocal cord palsy, stridor | yes, mild ataxia | yes, oculomotor paresis in all directions except downwards | no | no | no | yes, mandibular myoclonus | no | yes, mild ataxia | no | no | IVIg with improvement | DRB1*01:01/DRB1*04:04, **DQB1*05:01**/DQB1*03:02, HLA-A*02:01/HLA-A*03:01, HLA-B*07:02/HLA-B*18:01, HLA-C*03:04/HLA-C*07:01, DQA1*01:01/DQA1*03:01, DPB1*04:01/DPB1*04:01, DRB4*01:03, HLA-E*01:01/HLA-E*01:03, HLA-F*01:01/HLA-F*01:03, HLA-G*01:01/HLA-G*01:01 |
| 18 | 3 | male | 50 | 65 | 180 | chronic | bulbar syndrome, sleep disorder | n/a | yes, atypical movements | yes, OSAS | no | yes, dysphagia, dysarthria, bilateral vocal cord palsy | yes, mild gait ataxia | yes, horizontal gaze palsy, ptosis of the left eyelid | no | no | no | yes, oro-facio-mandibular dystonia | no | yes, mild ataxia | yes, mild tetraparesis, fasciculations on both upper and lower extremities | yes, spasticity and positive Babinski's sign in lower right extremity | IVMP, PLEX, RTX with improvement | **DRB1*10:01; DQB1*05:01** |
| 19 | 3 (PSP) | male | 67 | 76 | 108 | chronic | PSP-like syndrome, bulbar syndrome | n/a | no | yes, central apnea | yes, insomnia | yes, dysarthria, dysphagia, stridor | yes, axial instability | yes, hypometric horizontal saccades and nystagmus with slowed downgaze saccades | yes, mild cognitive impairment | yes, enuresis | no | no | yes, mild facial myokymia | yes, limb ataxia | no | yes, depression | no | DRB1*01:02, DRB1*03:01; DQB1*02:01, **DQB1*05:01** |
| 20 | 3 +TDP | female | 76 | 76 | 6 | subacute | bulbar syndrome | 4 | yes, sleep vocalizations, jerky movements | yes, acute respiratory failure, stridor | no | dysphagia, dysarthria, vocal cord paresis (stridor during sleep), acute respiratory failure (unclear if central hypoventilation or related to obstruction-vocal cord palsy; two ICU with ventilation required, finally underwent tracheostomy) | yes, shortstepped and slightly wide-based gait, postural instablity, unable to walk | yes, saccadic intrusions on pursuit eye movements | no | no | no | no | no | no | no | no | three cycles of intravenous and oral steroids; two cycles of cyclophosphamide | **DRB1*10:01, DQB1*05:01** |
| 21 | 3 +TDP | female | 66 | 71 | 60 | chronic | bulbar syndrome, possible sleep disorder | n/a | possible, during previous admission 'restlessness' during sleep | unknown | no | dsyphagia/aphagia, stridor, dysarthria | borderline abnormal tandem gait, reduced bilateral armswing | no | no | no | no | no | antecollis | no | no | no | no | unknown |
| 22 | 3 +TDP | female | 70 | 76 | 72 | chronic | bulbar syndrome, sleep disorder | n/a | yes, REM sleep disturbance | yes, OSAS, central apnea | yes, restless legs syndrom | yes, dysphagia | no | no | no | yes, recurrent  orthostatic vertigo | no | no | no | no | no | no | 2x IVIg, RTX, IVMP, PLEX | DRB1*01:01, DRB1*11:01, DQB1*03:01 **DQB1*05:01,** DQA1*01:01, DQA1*05:05 |
