# Supplementary material for: Neuropathological spectrum of anti-IgLON5 disease and stages of brainstem tau pathology: updated neuropathological research criteria of the disease-related tauopathy
Source: Acta Neuropathol. 2024 Oct 14;148(1):53. doi: 10.1007/s00401-024-02805-y (PMC11473580; doi:10.1007/s00401-024-02805-y)
Supplement: Supplementary file 9 — Supplementary Table 3: Details of the immunohistochemical stainings that have been performed in the different brain areas for the analysed cases. Numbers represent the case number in Tables 1 and 2 (DOCX 15 KB) [file 401_2024_2805_MOESM9_ESM.docx]

**Supplementary Table 3**

|  | **frontal ctx** | **motor ctx** | **temporal ctx** | **parietal ctx** | **occipital ctx** | **striatum** | **lenticular + NBM** | **ant/med hypothalamus** | **corp mamill** | **thalamus** | **amygdala** | **hippocampus + parahippocampal gyrus** | **cingulum** | **midbrain** | **pons** | **medulla** | **spinal cord** | **dentate cbl** | **cbl ctx** |
| --- | --- | --- | --- | --- | --- | --- | --- | --- | --- | --- | --- | --- | --- | --- | --- | --- | --- | --- | --- |
| **AT8** | 1,2,3,5,4,6,8,9,10,11,12,13,14,15,16,17,18,20,21,22 | 1,2,4,7,8,9,15,22 | 1,2,3,4,5,7,8,9,10,11,13,14,15,16,17,18,21,22 | 1,2,4,5,7,8,9,10,12,13,15,17,18,20,21,22 | 1,2,4,7,8,9,10,12,13,15,16,17,18,20,21,22 | 2,3,4,5,7,8,9,10,12,13,14,15,16,17,18,19,20,21,22 | 1,2,3,5,4,6,7,8,9,10,11,12,13,14,15,16,17,18,20,21,22 | 1,2,3,4,5,6,7,8,9,11,12,13,15,16,17,18,20,21,22 | 2,4,5,6,8,9,11,12,15,16,17,20,21,22 | 1,2,3,4,7,8,9,10,11,12,15,16,17,18,19,20,21,22 | 1,2,3,4,5,7,8,9,10,12,13,14,15,16,17,18,20,21,22 | 1,2,3,4,5,6,8,7,9,10,11,12,13,14,15,16,17,18,20,21,22 | 1,2,7,8,10,13,14,15,16,17,21,22 | 1,2,3,4,5,6,7,8,9,10,11,12,13,14,15,16,17,18,19,20,21,22 | 1,2,3,4,5,6,7,8,9,10,11,12,13,14,15,16,17,18,19,20,21,22 | 1,2,3,4,5,6,7,8,9,10,11,12,13,14,15,16,17,18,19,20,21,22 | 2,5,6,8,9,11,12,14,15,16,17,18,20,21 | 1,2,4,3,6,8,7,9,10,11,12,13,14,15,16,17,18,20,21,22 | 1,2,3,6,7,8,9,10,11,12,13,14,15,16,17,18,20,21,22 |
| **RD3** | 4,15,16 | 15 | 15,16 |  | 15,16 | 12,15,16,19,20 | 2,4,15,16 | 1,2,8,9,10,15,16,18,21 | 15,16 | 15,16 | 7,8,15,16 | 1,2,3,4,5,7,8,9,10,12,15,16,18,20,21,22 | 15,16 | 1,2,8,10,12,15,16,18,19,20,21,22 | 1,2,5,7,8,9,10,12,15,16,19,20,21,22 | 2,4,7,8,9,10,12,15,18,19,20,21,22 | 8,9,15,16,18 | 15,16 | 8,15,16 |
| **RD4** | 4,15,16 | 15 | 15,16 |  | 15,16 | 12,20,15,16,19 | 2,4,15,16,18 | 1,2,8,9,10,15,16,18,21 | 15,16 | 15,16 | 7,8,15,16 | 1,2,3,4,5,7,8,9,10,12,15,16,18,20,21,22 | 15,16 | 1,2,8,10,12,15,16,18,19,20,21,22 | 1,2,5,7,8,9,10,12,15,16,19,20,21,22 | 2,4,7,9,10,12,15,18,19,20,21,22 | 8,9,15,16,18 | 15,16 | 8,15,16 |
| **ßA4** | 1,2,3,4,5,6,7,8,9,10,11,12,13,14,15,16,17,18,20,21,22 | 2,4,15,22 | 2,3,4,5,8.9,10,12,11,13,14,16,15,18,20,22 | 1,2,3,4,5,9,10,12,13,14,15,16,18,20,21,22 | 2,4,8,9,12,15,18,20,21,22 | 1,2,3,4,7,9,10,12,16,18,19,20,21,22 | 15,16 | 4,8,12,15,16,20 | 15 | 4,12,20,21 | 1,2,3,4,7,8,9,10,12,15,16,18,20,21,22 | 1,2,3,4,5,6,7,8,9,10,11,12,13,14,15,17,18,20,21,22 | 15,16 | 1,2,3,4,6,7,8,9,10,12,15,18,19,20,21 | 16,19 | 15,19 | 15 | 6,15,16 | 1,2,3,4,6,7,8,9,10,12,15,16,18,20,21,22 |
| **a-syn** | 2,3,6,10,15,16,17 |  | 16 | 16,18 | 16 | 16 | 2,3,4,8,9,10,12,15,16,18,20 | 2,9,15,16 | 16 | 16 | 2,3,4,5,7,8,9,10,12,15,16,18,20,21,22 | 6,15,16,17,18,22 | 2,4,8,10,15,16,18 | 2,3,4,5,6,8,7,11,12,15,16,17,18,19,20,22 | 2,4,8,12,15,16,18,20,22 | 1,2,3,4,5,7,8,9,10,12,15,16,18,19,20,21,22 | 16 | 11,16 | 11,16 |
| **pTDP-43** | 1,2,3,4,5,6,8,9,10,12,13,15,16,17,18,20,21,22 | 1,2,6,7,9,11,21,22 | 1,2,4,8,9,15,16,21 | 2,8,10,21,22 | 8,15,16,21 | 2,3,5,8,9,10,15,18,19,21 | 2,5,6,8,9,10,15,16,17,18,19,22 | 1,2,4,5,6,7,8,9,10,12,15,16,17,20,21,22 | 2,6,8,15,16,17,21 | 2,4,5,8,10,15,17,18,19,21 | 1,2,3,4,7,8,9,10,15,18,22 | 1,2,3,4,5,6,7,8,9,10,11,13,15,16,17,18,21,22 | 8,15 | 1,2,3,4,5,6,7,8,9,10,12,15,16,17,19,20,21,22 | 1,2,4,5,6,7,8,9,10,12,15,16,17,18,19,20,21,22 | 1,2,3,4,5,6,7,8,9,10,11,13,15,16,17,18,19,21,22 | 2,5,6,8,9,10,11,15,16,17,18,21 | 2,6,8,9,10,15,16,17,18,21,22 | 2,6,8,10,15,16,17,18,21,22 |
| **p62/ubiquitin** | 1,2,4,6,8,9,10,12,15,17,18,20,21,22 |  |  | 16,21 |  | 21 | 2,15,16,21 | 2,16,21 |  | 21 | 16,21 | 1,2,4,5,6,8,9,10,12,15,17,18,20,21,22 | 16 | 6,10,17,21 | 2,10,16,21 | 2,10,15,21 | 2,6,16 | 6,8,15,16,21 | 1,2,4,6,8,9,10,12,15,16,7,18,20,21,22 |
